# FGFR blockade boosts T cell infiltration into triple-negative breast cancer by regulating cancer-associated fibroblasts

**DOI:** 10.7150/thno.68972

**Published:** 2022-05-27

**Authors:** Yushen Wu, Ziying Yi, Jie Li, Yuxian Wei, Rui Feng, Jiazhou Liu, Jiefeng Huang, Yuru Chen, Xiaoyu Wang, Jiazheng Sun, Xuedong Yin, Yunhai Li, Jingyuan Wan, Li Zhang, Jing Huang, Huimin Du, Xiaoyi Wang, Qin Li, Guosheng Ren, Hongzhong Li

**Affiliations:** 1 Chongqing Key Laboratory of Molecular Oncology and Epigenetics, The First Affiliated Hospital of Chongqing Medical University; Chongqing, 400016, China.; 2 Department of Endocrine and Breast Surgery, The First Affiliated Hospital of Chongqing Medical University; Chongqing, 400016, China.; 3 Department of Breast and Thyroid Surgery, Chongqing General Hospital, Chongqing, 401147, China.; 4 Department of Pharmacology, Chongqing Medical University; Chongqing, 400016, China.; 5 Department of Pathophysiology, Chongqing Medical University; Chongqing, 400016, China; 6 Department of Respiratory, The First Affiliated Hospital of Chongqing Medical University; Chongqing, 400016, China.; 7 Department of Oncology, The First Affiliated Hospital of Chongqing Medical University; Chongqing, 400016, China.; 8 Department of Oncology, Beijing Friendship Hospital, Capital Medical University; Beijing, 100050, China.; 9 Department of Dermatology, Shenzhen People's Hospital (The Second Clinical Medical College, Jinan University; The First Affiliated Hospital, Southern University of Science and Technology); Shenzhen, 518020, China.

**Keywords:** FGFR, breast cancer, fibroblast, VCAM-1, immunotherapy

## Abstract

**Background:** Since T cell exclusion contributes to tumor immune evasion and immunotherapy resistance, how to improve T cell infiltration into solid tumors becomes an urgent challenge.

**Methods:** We employed deep learning to profile the tumor immune microenvironment (TIME) in triple negative breast cancer (TNBC) samples from TCGA datasets and noticed that fibroblast growth factor receptor (FGFR) signaling pathways were enriched in the immune-excluded phenotype of TNBC. Erdafitinib, a selective FGFR inhibitor, was then used to investigate the effect of FGFR blockade on TIME landscape of TNBC syngeneic mouse models by flow cytometry, mass cytometry (CyTOF) and RNA sequencing. Cell Counting Kit-8 (CCK-8) assay and transwell migration assay were carried out to detect the effect of FGFR blockade on cell proliferation and migration, respectively. Cytokine array, western blot, enzyme-linked immunosorbent assay (ELISA) and immunofluorescence (IF) were employed to investigate the potential mechanism by which FGFR inhibition enhanced T cell infiltration.

**Results:** Blocking FGFR pathway by Erdafitinib markedly suppressed tumor growth with increased T cell infiltration in immunocompetent mouse models of TNBC. Mechanistically, FGFR blockade inhibited cancer-associated fibroblasts (CAFs) proliferation, migration and secretion of vascular cell adhesion molecule 1 (VCAM-1) by down-regulating MAPK/ERK pathway in CAFs, thus promoting T cell infiltration by breaking physical and chemical barriers built by CAFs in TIME. Furthermore, we observed that FGFR inhibition combined with immune checkpoint blockade therapy (ICT) greatly improved the therapeutic response of TNBC tumor models.

**Conclusions:** FGFR blockade enhanced ICT response by turning immune “cold” tumor into “hot” tumor, providing remarkable implications of FGFR inhibitors as adjuvant agents for combinatorial immunotherapy.

## Introduction

T cells in the tumor microenvironment (TME) play a key role in cancer immune surveillance. Generally, higher T cell infiltration into tumors predicts better survival for patients with malignant tumors 1, 2. Current cancer immunotherapy by immune checkpoint blockade therapy (ICT) has been harnessing this fact to unleash the power of T cells by eradicating negative signals such as cytotoxic T lymphocyte associated antigen-4 (CTLA-4), and programmed death-1 (PD-1)/programmed death ligand-1 (PD-L1) that hinder T cell function 1, 3. Although ICT has shown unprecedented clinical activities in a variety of cancers, only a minority of treated patients respond. Multiple elements contribute to ICT resistance, including tumor mutation burden (TMB) level, the degree of cytotoxic T cell (CTL) infiltration, PD-L1 level, immunosuppressive factors in TME, and gut bacteria 4, 5. Increasing studies focus on combination approaches to eliminate the factors inducing ICT resistance and further improve therapeutic efficacy 6, 7. Regardless of ICT itself or combination therapies, the ultimate aim is to boost T cell-mediated cytotoxic/cytolytic activity. Therefore, the presence of T cells in the tumor parenchyma would be a critical prerequisite for optimal therapeutic effects 8.

Based on the histological examination of tumor biopsies, three major immune-associated phenotypes of TME have been identified in solid cancers, namely the immune-inflamed, immune-excluded, and immune-desert phenotypes 1, 9. The immune-inflamed subtype and immune-desert subtype are characterized by abundant T cell infiltration and scarce T cell infiltration into tumors especially tumor parenchyma, respectively. Intricately, although a large number of immune cells exist in tumors of immune-excluded subtype, these immune cells do not penetrate the tumor parenchyma but instead are imprisoned in the stroma that surrounds the cancer nest. The immune-desert subtype and the immune-excluded subtype are both regarded as non-inflamed “cold” tumors 1. Exploring the molecular and cellular basis for the differences among these immune phenotypes will help to unravel the underlying mechanisms involved in tumor immune escape and immunotherapy resistance.

Breast cancer remains a major public health problem worldwide, accounting for 30% of all cancers in women and 15% of female cancer-related deaths 10. Clinically, breast tumors are classified into hormone receptor positive (HR^+^) tumors expressing the estrogen (ER) and/or progesterone (PR) receptors, human epidermal receptor 2 (HER2)-enriched tumors with HER2 overexpression in the absence of HR expression, and triple negative breast cancer (TNBC) lacking expression of all three receptors. Compared with non-TNBC, TNBC is an aggressive subtype with higher incidence of metastases, poorer prognosis and limited therapeutic options, standing for a vital challenge in clinical practice 11. Nowadays, growing evidence shows that TNBC is a more immunogenic subtype compared to other subtypes, suggesting that TNBC patients are easier to get benefit from immunotherapy. Nevertheless, single-agent efficacy of ICT in TNBC still remains limited 12. It was demonstrated that almost half of TNBC were immune-excluded and about a third were categorized as immune-desert type 13, indicating that turning tumor from immune “cold” to immune “hot”, especially reversing T cell exclusion in immune-excluded type, might improve anticancer immune response in TNBC. Here, we explored the immunological status of the TME in TNBC and identified that FGFR signaling pathway was enriched in immune-excluded phenotype compared to immune-inflamed phenotype. Therefore, we sought to test whether targeting FGFR pathway could promote T cell infiltration and synergize with current ICT.

## Results

### FGFR activation is correlated with T cell exclusion in TNBC

**Figure 1:**
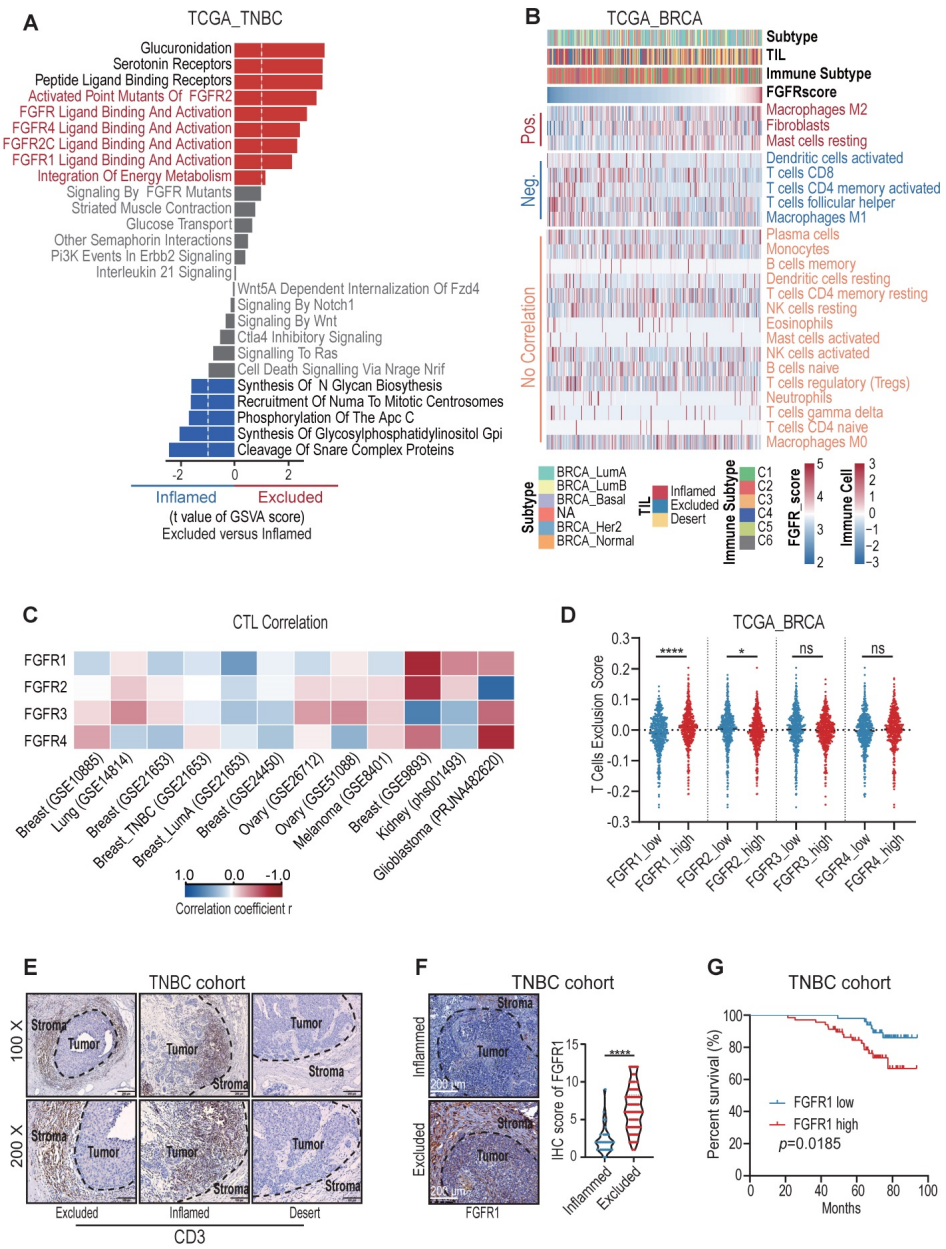
FGFR signaling pathways were enriched in immune-excluded type triple-negative breast cancer (TNBC). A) The gene signature in immune-inflamed and immune-excluded TNBC samples from TCGA dataset. B) The correlation between FGFR score and 23 types of stromal cells in TME based on TCGA BRCA dataset. “Pos.” represents immune cells positively correlated with FGFR score; “Neg.” represents immune cells negatively correlated with FGFR score; “No correlation” represents immune cells that do not correlate with FGFR score. Immune subtypes (C1-C6) were characterized by differences in the nature of the overall immune response^14^.C) The correlation between FGFR1/2/3/4 expression and cytotoxic T lymphocytes (CTL) infiltration in indicated cancer types from GEO database based on Tumor Immune Dysfunction and Exclusion (TIDE) system. D) T cell exclusion score in BRCA of TCGA based on FGFRs expression. E) Immune phenotypes of TNBC defined by IHC staining of CD3. F) The expression of FGFR1 in immune-inflamed and immune-excluded TNBC samples based on IHC staining (inflamed, n=33; excluded, n=118, t test). G) Kaplan-Meier survival analysis of low FGFR1 (blue, n=51) versus high FGFR1 (red, n=68) expression in TNBC.

We first employed deep learning to profile the tumor immune microenvironment (TIME) in TNBC samples from TCGA dataset 14, and we noticed that about one-third of TNBC were categorized as immune-excluded phenotype (Figure S1A). In order to clarify the key factors prompting T cell exclusion, we retrieved the mRNAseq data from the TCGA dataset and compared the gene signature between immune-excluded and immune-inflamed TNBC samples. Notably, the significant activation of FGFR pathways was enriched in immune-excluded group (Figure 1A). FGFR score was then calculated based on the expression values of FGFR1-4. BRCA tumors with high FGFR score had lower CD8^+^ T cell infiltration and higher T cell exclusion score (Figure S1C-D) 15. Next, we further analyzed the correlation between FGFR and 23 types of stromal cells in TME of breast cancer samples. It showed that FGFR was negatively correlated with CD8^+^ T cell and M1 macrophage, and positively associated with fibroblast and M2 macrophage (Figure 1B).

Furthermore, we found that FGFRs were negatively correlated with cytotoxic T lymphocytes (CTL) infiltration in multiple cancer types including breast, colorectal, head neck, lung, skin and ovarian cancers based on Tumor Immune Dysfunction and Exclusion (TIDE) system (Figure 1C, Figure S1E). Remarkedly, FGFR1 was more negatively correlated with T cell infiltration, compared with other FGFRs (Figure 1C-D) 15. We then characterized the immunophenotypes in the samples from a TNBC cohort, and observed that more than half of TNBC included were classified as immune-excluded phenotype based on CD3 immunohistochemical (IHC) staining in TME (Figure 1E, Figure S1B), which is consistent with previous study 13. IHC staining of FGFR1 in this TNBC cohort also demonstrated higher expression of FGFR1 in immune-excluded group compared with immune-inflamed group (Figure 1F). These observations above suggest that FGFR pathways were associated with T cell exclusion. Moreover, we found that high FGFR expression was correlated with shorter overall survival of TNBC patients (Figure 1G), which was also validated in the Kaplan-Meier plotter database (Figure S1F) 16.

### Blocking FGFR enhanced T cell infiltration into TNBC *in vivo*

**Figure 2:**
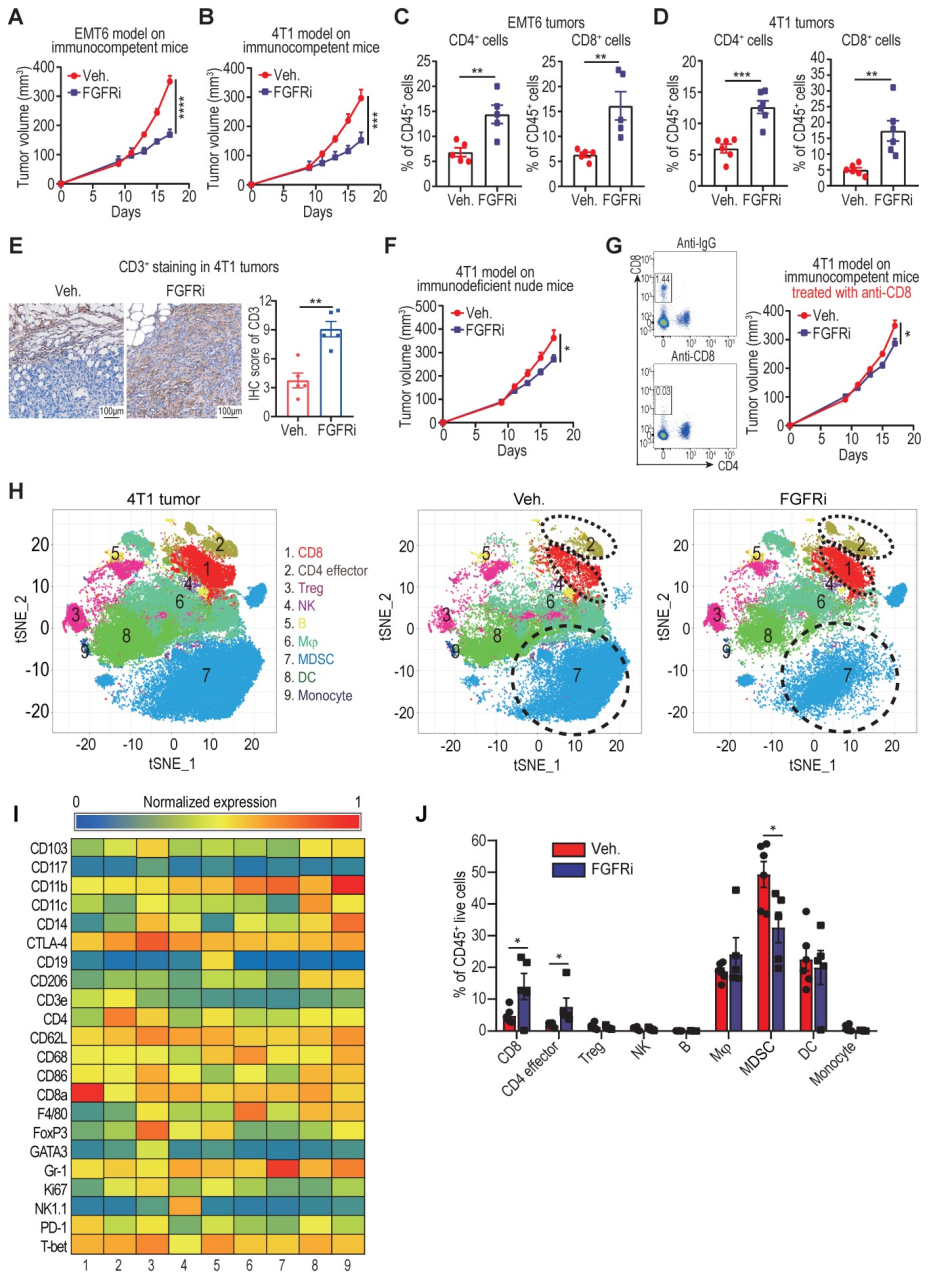
Blocking FGFR signaling pathway promoted T cell infiltration into TNBC. A and B) EMT6 (A) and 4T1 (B) tumor growth in vehicle-treated *versus* FGFR inhibitor (FGFRi) Erdafitinib-treated immunocompetent BALB/c mice (n=7 mice/group, two-way ANOVA). C and D) Percentages of CD4^+^ and CD8^+^ T cells in primary EMT6 (C) and 4T1 (D) tumors from vehicle-treated *versus* FGFRi-treated mice (n=6, t test). E) Representative IHC staining of CD3 in tumor tissues from vehicle-treated *versus* FGFRi-treated mice. F) 4T1 tumor growth in vehicle-treated *versus* FGFRi-treated immunodeficient nude mice (n=7 mice/group, two-way ANOVA). G) 4T1 tumor growth in vehicle-treated *versus* FGFRi-treated BALB/c mice where CD8^+^ T-cells were depleted by anti-CD8 antibodies (n=6 mice/group, two-way ANOVA). H) t-distributed stochastic neighbor embedding (tSNE) plot of tumor-infiltrating leukocytes overlaid with color-coded clusters in 4T1 tumors from vehicle-treated *versus* FGFRi-treated BALB/c mice. Dotted ellipses highlight clusters with significant differences between two groups. I) Heat map displaying normalized marker expression of each immune cluster. J) Frequency of clusters of indicated immune cell subsets. Data are mean ± s.e.m. (n=5 mice/group, t test).

We employed TNBC syngeneic mouse models (EMT6 and 4T1 cell lines) to further investigate the relationship between FGFR and T cell infiltration. Constraining FGFR pathways by a selective FGFR inhibitor (FGFRi) Erdafitinib significantly suppressed tumor growth with increased CD3^+^ (CD4^+^ and CD8^+^) T cell infiltration in immunocompetent BALB/c mice bearing EMT6 or 4T1 tumors (Figure 2A-E, Figure S2A). Given that FGFRi was reported to suppress tumor cell proliferation 17, 18, we wonder about the contribution of FGFRi-induced T cell infiltration to the observed tumor shrinkage. Thus, we next used immunocompromised mice to test the anti-tumor activity of FGFRi. When CD8^+^ T cells were lacking in athymic nude mice or depleted by anti-CD8 antibodies in BALB/c mice, the suppression of FGFRi on tumor growth was significantly alleviated (Figure 2F-G), showing that FGFRi-mediated anti-tumor activity partly depends on CD8^+^ T cells. To evaluate the global impact of FGFR blockade on TIME, we profiled CD45^+^ immune cells from 4T1 tumors in vehicle- or FGFRi-treated mice using CyTOF (Figure 2H-I, Figure S2B). 4T1 tumors from FGFRi-treated mice had significantly increased CD8^+^ T cells (cluster 1) and CD4^+^ T cells (cluster 2), and decreased myeloid-derived suppressor cells (MDSC) (cluster 7), suggesting enhanced anti-tumor immunity (Figure 2J).

### FGFR blockade induced T cell infiltration by modulating fibroblasts

**Figure 3:**
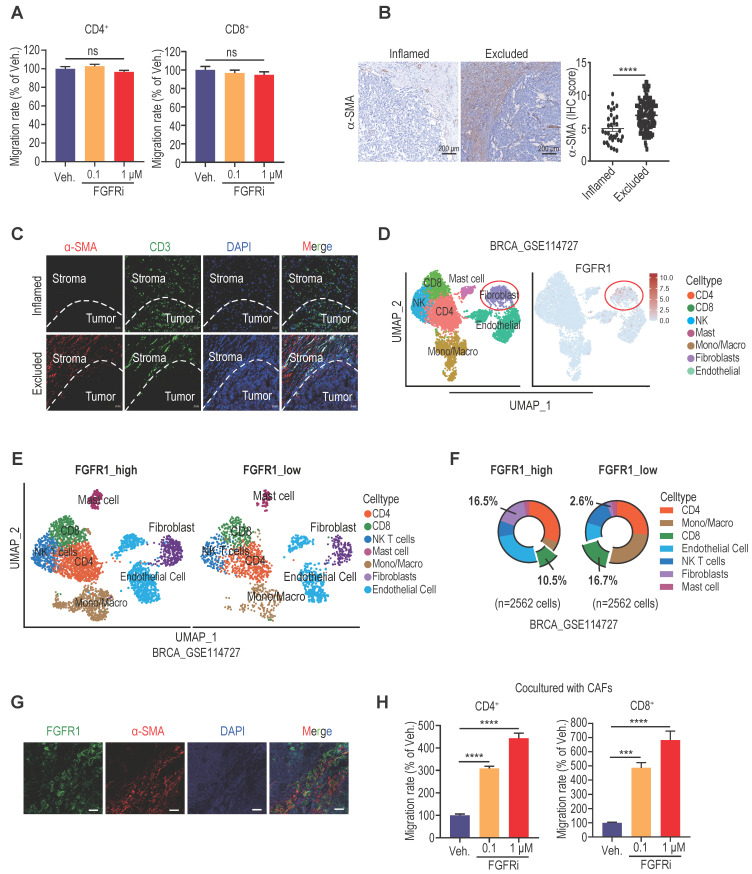
FGFR blockade induced T cell infiltration by modulating fibroblasts. A) The effect of FGFRi Erdafitinib on CD4^+^ and CD8^+^ T cell migration was detected by transwell migration assay (n=5 biological replicates, one-way ANOVA). B) Representative staining and IHC score of α-SMA in immune-inflamed and immune-excluded TNBC samples. C) Representative IF staining of α-SMA and CD3 in immune-inflamed and immune-excluded TNBC samples. D) FGFR1 expression in tumor microenvironment of breast cancer (GSE114727). E-F) Cell population in TME of breast cancer based on FGFR1 expression. G) Representative IF staining of FGFR1 and α-SMA in TNBC samples. H) The effect of FGFRi Erdafitinib on CD4^+^ and CD8^+^ T cell migration in presence of CAFs was detected by transwell migration assay (n=3 biological replicates, one-way ANOVA).

Since T-cell motility contributes to T cell infiltration into tumors 2, 19, we next detected the direct effect of FGFR blockade on T cell motility. However, transwell migration assay showed no significant change in splenic T cell migration after Erdafitinib treatment *in vitro* (Figure 3A), suggesting that FGFR indirectly affects T cell infiltration. Among stromal cells in TME, FGFRs were significantly correlated with fibroblasts (Figure S3A). Increasing evidence highlighted a critical role of cancer-associated fibroblasts (CAFs) in promoting T cell exclusion 20,21. We next confirmed that a large number of CAFs were distributed in immune-excluded TNBC tumors by IHC staining of fibroblast marker α-SMA (Figure 3B). Moreover, double immunofluorescence (IF) staining of α-SMA and CD3 in immune-excluded tumors demonstrated that CAFs are mostly distributed in the periphery of cancer nests, shielding tumor cells from T cell attack (Figure 3C). Based on the Tumor Immunity Single Cell Center (TISCH) database, we revealed that FGFR1 is mainly expressed on fibroblasts in TME of breast cancer (Figure 3D, Figure S3B-C). Furthermore, breast cancer samples were grouped according to FGFR1 expression in a single-cell dataset (GSE114727), and it also showed that the group with high FGFR1 expression had more fibroblasts and less CD8^+^ T cell infiltration in TME (Figure 3E, 3F). In agreement, the dominant expression of FGFR1 on CAFs was validated by double IF staining of FGFR1 and α-SMA on human TNBC samples (Figure 3G). To explore whether FGFRs on CAFs mediate T cell exclusion, we performed the transwell migration assay by co-culturing splenic T cells and vehicle- or FGFRi-treated CAFs (Figure 3H, Figure S3D). Remarkably, FGFRi significantly enhanced T cell migration in the presence of CAFs (Figure 3H), indicating that FGFR blockade improves T cell infiltration mainly via modulating CAFs.

### Inhibiting FGFR suppressed CAFs proliferation, migration and VCAM-1 secretion

CAFs are a key component of the tumor stroma, where they cool down the immune microenvironment as a physical barrier and source of immunosuppressive molecules 20, 21. To further dig out the mechanism responsible for FGFRs-mediated T cell exclusion, we then examined the detailed biological effects of FGFR blockade on CAFs.

**Figure 4:**
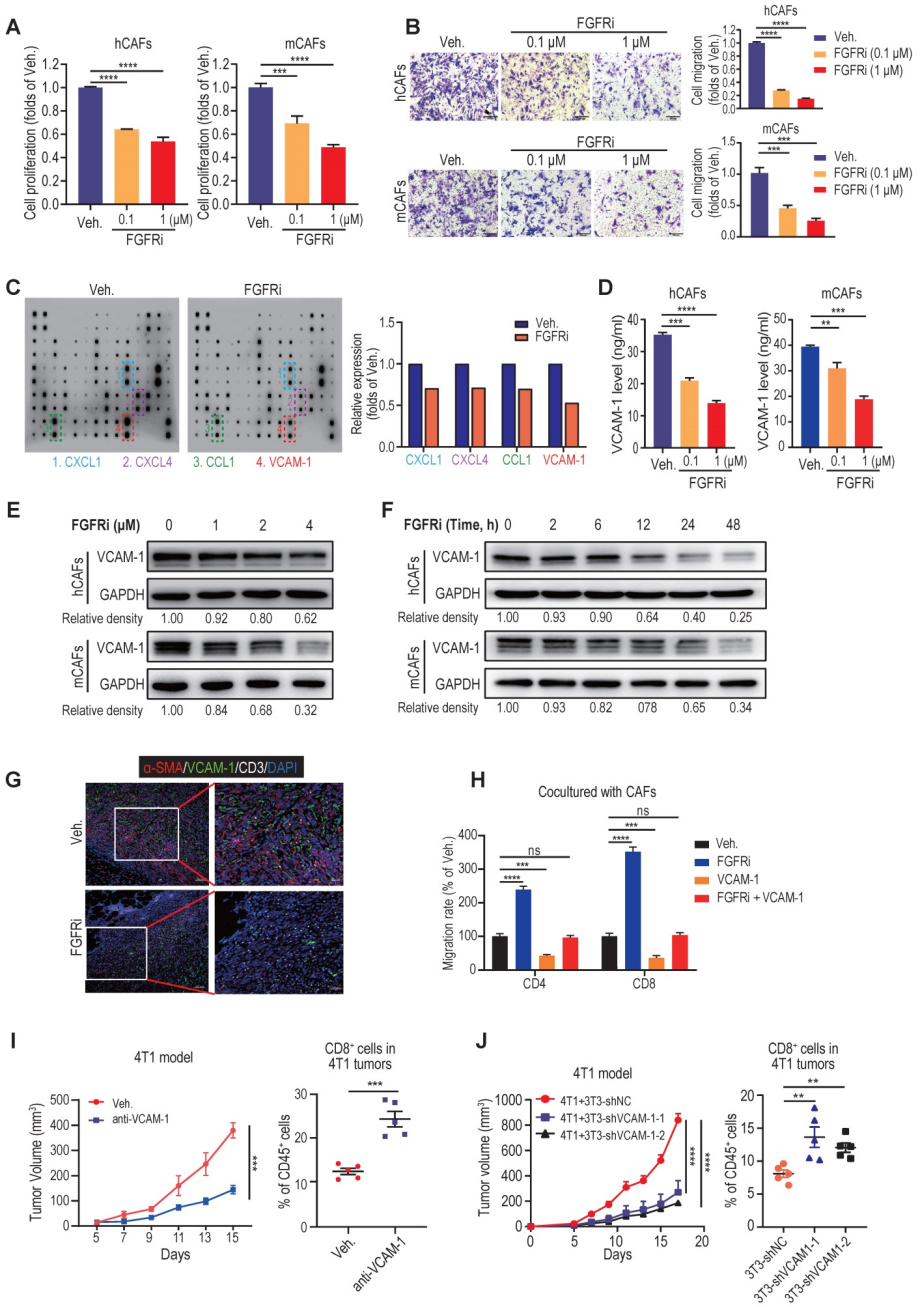
Blocking FGFR pathway inhibited cell proliferation, migration and VCAM-1 secretion of CAFs. A) The effect of FGFRi Erdafitinib on cell proliferation of human CAFs and mouse CAFs for 48 h was detected by CCK-8 assay (n=3 biological replicates, one-way ANOVA). B) The effect of FGFRi Erdafitinib on cell migration of human CAFs and mouse CAFs was detected by transwell migration assay (n=3 biological replicates, one-way ANOVA). C) Cytokine arrays for vehicle-treated *versus* FGFRi-treated mouse CAFs. Boxes indicate the cytokines with significant changes. D) The effect of FGFRi Erdafitinib on VCAM-1 level in cell supernatant of human CAFs and mouse CAFs was detected by ELISA (n=3 biological replicates, one-way ANOVA). E) The effect of different concentrations of FGFRi Erdafitinib on VCAM-1 expression in human CAFs and mouse CAFs was examined by western blot. F) The effect of different durations of FGFRi Erdafitinib on VCAM-1 expression in human CAFs and mouse CAFs was examined by western blot. G) Representative IF staining of α-SMA, VCAM-1 and CD3 in 4T1 tumors from vehicle- and FGFRi-treated mice. H) The effect of recombinant VCAM-1 (10 μM) or/and Erdafitinib (1 μM) on CD4^+^ and CD8^+^ T cell migration in presence of CAFs was detected by transwell migration assay (n=3 biological replicates, one-way ANOVA). I) 4T1 tumor growth and CD8^+^ T cell infiltration in tumors of BALB/c mice treated with vehicle or anti-VCAM1 antibody (n=5 mice/group, two-way ANOVA). J) 4T1 tumor growth and CD8^+^ T cell infiltration in tumors of BALB/c mice. 4T1 cells were co-transplanted with 3T3 shNC control cells or 3T3 shVCAM1 cells (n=5 mice/group, two-way ANOVA).

Cell Counting Kit-8 (CCK-8) assays demonstrated that FGFRi Erdafitinib significantly inhibited cell proliferation of human CAFs (hCAFs), mouse CAFs (mCAFs) and 3T3 mouse fibroblasts at concentrations of 0.1 and 1 μM (Figure 4A, Figure S4A), whereas FGFRi did not have a dramatic impact on TNBC cell proliferation at the same concentrations (Figure S4B). In addition, transwell migration assay showed that FGFRi markedly suppressed fibroblasts migration (Figure 4B, Figure S4C). Collectively, these observations advocated that the activation of FGFR has the potential to help CAFs build physical “fences” by promoting CAFs proliferation and migration. Next, in order to elucidate whether FGFRs also prompt T cell exclusion by producing immunosuppressive factors, we compared the cytokine profiles secreted by vehicle-treated and FGFRi-treated mCAFs using antibody microarrays and identified a panel of cytokines regulated by FGFRi (Figure 4C). Among them, vascular cell adhesion molecule-1 (VCAM-1), which was reported to augment tumor immune evasion and found dominantly expressed in CAFs (Figure S4D) 22, 23, ranked as one of the top cytokines down-regulated by FGFRi (Figure 4C). Indeed, the inhibitory effect of FGFRi on VCAM-1 in hCAFs, mCAFs and 3T3 cells was validated by ELISA and western blot (Figure 4D-E, Figure S4E-F). The restraint of FGFRi to VCAM-1 was gradually strengthened with the prolongation of treatment time (Figure 4F, S4G). Consistently, the decreased VCAM-1 level in 4T1 and EMT-6 tumors of FGFRi-treated mice was detected by ELISA (Figure S4H). Additionally, triple IF staining demonstrated a smaller number of CAFs, decreased VCAM-1 expression and increased CD3^+^ T cell infiltration in 4T1 tumors of FGFRi-treated mice (Figure 4G). Adding VCAM-1 significantly attenuated the promotion of T cell migration by FGFRi in the presence of CAFs (Figure 4H). Furthermore, *in vivo* use of anti-VCAM1 neutralizing antibodies also significantly inhibited tumor growth with enhanced T cell infiltration (Figure 4I, Figure S4I). Co-transplantation of VCAM-1-knockdown 3T3 fibroblasts with 4T1 cells into BALB/c mice resulted in markedly reduced tumor growth with significantly increased tumor-infiltrating T-cells, compared to co-transplanting control 3T3 fibroblasts with 4T1 cells (Figure 4J, Figure S4J). Together, these results indicate that blocking FGFR signaling pathways might promote T cell infiltration by disrupting the physical barrier built by CAFs and inhibiting the secretion of VCAM-1 from CAFs.

### MAPK/ERK activation maintains FGFR function in CAFs *in vitro*

**Figure 5:**
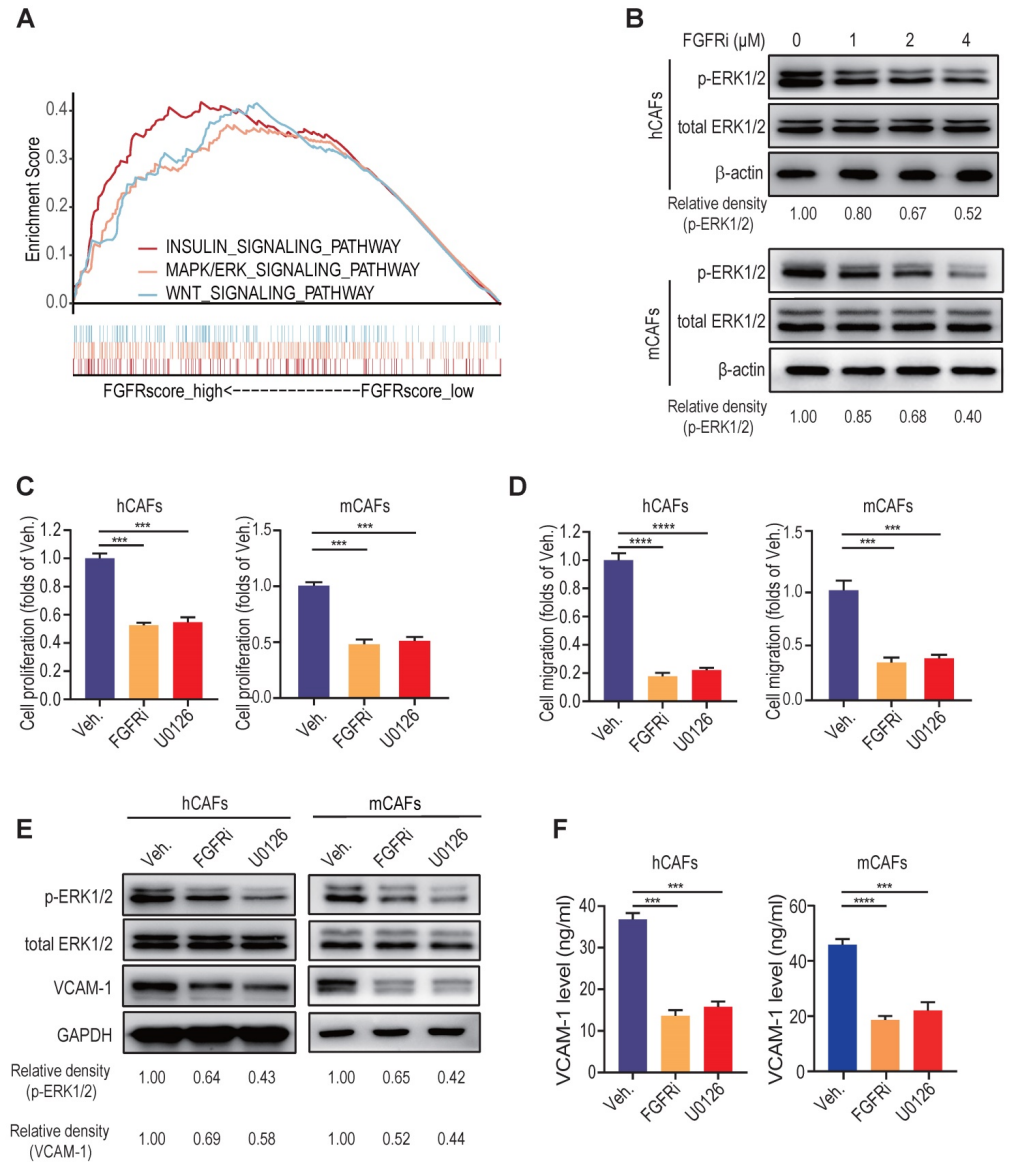
MAPK/ERK activation maintains FGFR function in CAFs *in vitro*. A) GSEA (Reactome pathway analysis) of FGFR score in breast cancer from GEO. B) The effect of FGFRi Erdafitinib on p-ERK1/2 and total ERK1/2 expression of human CAFs and mouse CAFs was detected by western blot. C) The effect of MAPK pathway inhibitor U0126 on cell proliferation of human CAFs and mouse CAFs for 48 h was detected by CCK-8 assay (n=3 biological replicates, one-way ANOVA). D) The effect of U0126 on cell migration of human CAFs and mouse CAFs was detected by transwell migration assay (n=3 biological replicates, one-way ANOVA). E) The effect of U0126 on VCAM-1 expression of human CAFs and mouse CAFs was detected by western blot. F) The effect of U0126 on VCAM-1 level in cell supernatant of human CAFs and mouse CAFs was detected by ELISA (n=3 biological replicates, one-way ANOVA).

To unravel the signaling pathways concerning FGFR function, we conducted gene set enrichment analysis (GSEA) in breast cancer from GEO datasets. We found that FGFRs and MAPK/ERK signaling pathway are highly interrelated (Figure 5A, Figure S5A), which was then verified by western blot showing that FGFRi evidently down-regulated the activation of MAPK/ERK pathway in CAFs in a dose-dependent manner (Figure 5B, Figure S5B). Inhibiting ERK pathway by a selective inhibitor U0126 significantly suppressed the proliferation and migration of hCAFs, mCAFs and 3T3 fibroblasts (Figure 5C-D, Figure S5C-D). Moreover, U0126 considerably inhibited VCAM-1 expression and secretion on fibroblasts (Figure 5E-F, Figure S5E-F). These findings suggest that the activation of MAPK/ERK signaling pathway on CAFs might be responsible for sustaining FGFR function involved in the induction T cell exclusion.

### FGFR inhibition enhances therapeutic responses to ICT

**Figure 6:**
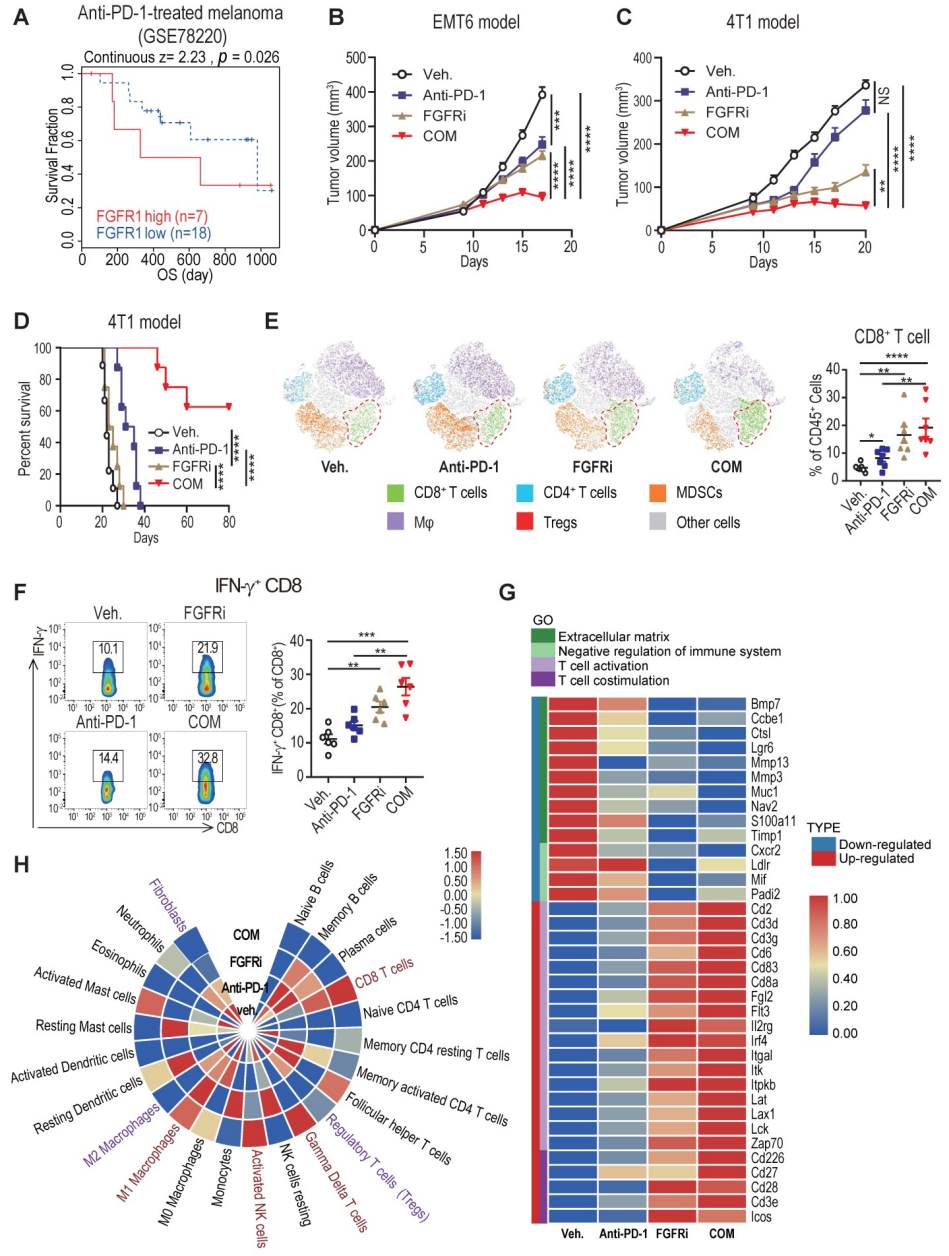
FGFR blockade synergizes with immune checkpoint blockade therapy. A) Overall survival of melanoma patients who had high FGFR1 vs. low FGFR1 expressed in the tumors before anti-PD-1 treatment (GSE78220). B and C) EMT6 (B) and 4T1 (C) tumor growth in mice treated with vehicle, anti-PD-1, FGFRi (Erdafitinib) or combination of anti-PD-1 and FGFRi (n=7 mice/group, two-way ANOVA). D) Survival analysis of 4T1 tumor-bearing mice treated with indicated therapy (n=8 mice/group, log-rank test). E) The t-SNE plot of TILs and CD8^+^ T cell population in 4T1 tumors from mice treated with indicated therapies (n=6, one-way ANOVA). F) Percentage of IFN-γ^+^ CD8^+^ T-cells in indicated therapy-treated 4T1 tumors (n=6, one-way ANOVA). G) Gene ontology (GO) analysis by RNA-seq of 4T1 tumors in indicated groups (n=3/group). Heatmap shows the DEGs and associated signatures. COM, anti-PD-1+FGFRi. H) Heatmap shows the percentage of tumor infiltrating immune cells and fibroblasts in indicated therapy-treated 4T1 tumors.

Accumulating studies strongly support that T cell exclusion contributes to immunotherapy resistance, and look into ways to turn immune “cold” tumors into “hot” tumors to reach higher responsiveness to ICT 2. To examine whether blocking FGFR pathway would reverse T cell exclusion to facilitate ICT for cancer patients, we further explored whether high FGFR expression is associated with immunotherapy resistance. Among melanoma patients treated with the anti-PD-1 drug pembrolizumab (GSE78220) 24, relatively higher FGFR1 mRNA expression was found in pre-treatment tumors from non-responding cases compared with that from responders (Figure S6A). The anti-PD-1-treated patients with high FGFR1-expressing tumors had dreadfully shorter overall survival compared with patients with low FGFR1-expressing tumors (Figure 6A). Consistently, the correlation between FGFR3 expression and poor progression-free survival of anti-PD-1-treated patients was also confirmed in another independent dataset (Figure S6B). To test if FGFR inhibition would enhance the anti-tumor activity of PD-1 blockade, 4T1 and EMT6 tumors were treated by vehicle, anti-PD-1 antibody, FGFRi, and combinatorial treatment of FGFRi plus anti-PD-1, respectively. We found that FGFRi treatment combined with ICT attained the highest therapeutic response compared with FGFRi or ICT alone in both ICT-resistant 4T1 model and ICT-sensitive EMT6 model (Figure 6B-C). Remarkably, complete tumor remission was observed in 60% of the combination treatment group but none of the other groups (Figure 6D), along with significantly increased CD4^+^ and CD8^+^ T cell infiltration, and decreased infiltration of myeloid-derived suppressor cells (MDSC), macrophages (Mφ) and regulatory T cells (Treg) (Figure 6E, Figure S6C-F).

Furthermore, increased infiltration of IFN-γ^+^ CD8^+^ T cells was detected in FGFRi-treated 4T1 tumors and further improved in FGFRi plus ICT combination group (Figure 6F), which suggests that boosted cytotoxic activities of CD8^+^ T cells also contribute to FGFRi blockade-mediated anti-tumor immunity. The FGFR-induced T cell dysfunction (down-regulated cytotoxic/cytolytic activity of CTLs) was also supported by the TIDE system-based analysis of the correlation between FGFRs and T cell dysfunction (Figure S6G-I). To gain deep insight into anti-tumor immunity of FGFR inhibition, we compared 4T1 tumors with different treatments for their global transcriptomic differences by RNA sequencing. Compared to vehicle control, FGFRi suppressed the expression of extracellular matrix synthesis related genes (e.g., BMP7, MMP13 and MMP3) which may help CAFs build the physical barrier (Figure 6G). The α-SMA expression was also significantly reduced in FGFRi-treated 4T1 tumors, indicating that FGFRi markedly inhibited CAFs infiltration and destroyed the physical barrier established by CAFs *in vivo* (Figure S6J). Additionally, genes involved in negative regulation of immune system were down-regulated in FGFRi-treated group, while those related to T cell activation and co-stimulation were up-regulated in this group (Figure 6G). More notably, the combination of therapies further strengthened the trend of these parameters (Figure 6G). Further analysis based on RNA-seq revealed noticeably increased CD8^+^ T cells, γδ T cells, activated NK cells and M1 Mφ, and decreased fibroblasts, Treg and M2 Mφ in combination group compared to vehicle control group (Figure 6H). Collectively, these studies showed that FGFR inhibition can synergize with ICT to promote anti-tumor immune responses.

## Discussion

Increasing evidence has uncovered two distinct mechanisms of tumor immune evasion, namely T cell exclusion and T cell dysfunction. In some tumors, immunosuppressive elements exclude T cells from infiltrating tumors. In other tumors, T cells tend to be in a dysfunctional state, despite the fact that a large number of T cells exist in these tumors 15. An adequate penetration into TME and successful activation of effector T cells have been recognized as a critical premise for ideal responses to T cell-based immunotherapies including ICT. However, tumor cells have utilized abundant mechanisms to block T cell access to the tumor tissue especially tumor nest 2, 4. In TME, CAFs constitute the major component of the tumor stroma, and play generally an immunosuppressive role via their surfaceome, matrisome, secretome, and metabolome. Accumulating studies show that CAFs contribute to T cell exclusion as a physical barrier and source of immunosuppressive molecules. CAFs and extracellular matrix (ECM) remodeled by CAFs tend to construct a physical “fence” to shield T cells from tumor nest. Additionally, the immunosuppressive cytokines and chemokines such as TGF-β and CXCL12 released by CAFs can also inhibit T cell proliferation, motility and even activation. However, the detailed mechanism involved in the regulation of CAFs-mediated pro-tumor immunity still remains enigmatic 20, 25, 26.

**Figure 7:**
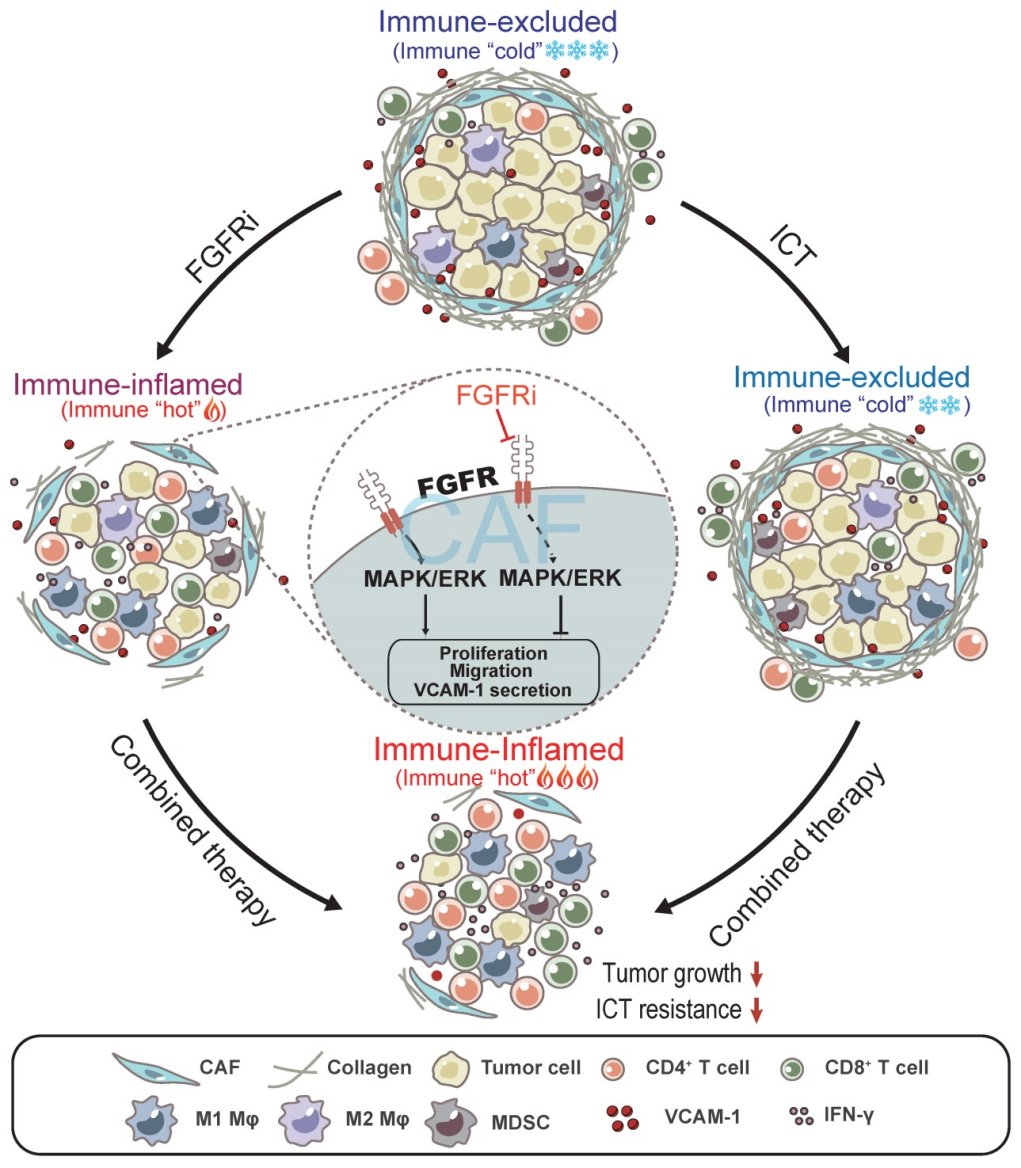
Model: FGFR blockade reverses T cell exclusion and ICT resistance by modulating CAFs. Blocking FGFR by FGFR inhibitor (FGFRi) suppresses the activation of MAPK/ERK signaling pathway in CAFs, thereby inhibiting the proliferation, migration and secretion of VCAM-1 of CAFs, leading to the breakage of physical and chemical barriers built by CAFs to prevent T cell infiltration. Notably, FGFRi improves ICT efficacy by increasing the infiltration of anti-tumor immune cells such as CD8^+^ T cells and M1 macrophages, inhibiting the infiltration of pro-tumor immune cells such as MDSC and M2 macrophages, and enhancing anti-tumor activity of CTLs in tumors.

In this study, we found that FGFR signaling pathways are enriched in immune-excluded phenotype of TNBC and contribute to T cell exclusion by modulating CAFs (Figure 7). FGFRs consisting of FGFR1-4 belong to the cell-face receptor tyrosine kinase (RTK) superfamily, triggering classic downstream signaling pathways such as MAPK signaling pathway to exert essential roles in comprehensive biological functions via binding to FGFs. FGFR gene amplification or/and mutation prompt malignant biological behaviors of tumor cells and stromal cells, such as cell survival, proliferation, invasion and differentiation 17, 27. Interestingly, a recent study showed that the combination of FGFR inhibition and anti-PD-1 drives expansion of T-cell clones and immunologic changes in the TME to improve anti-tumor immunity in lung cancer 28, suggesting that FGFR might also contribute to tumor progression via affecting anti-tumor immunity. However, the detailed mechanism regarding FGFR-mediated immune evasion in tumors especially TNBC still remains unclear. Given the importance of FGFR signaling pathway in progression of multiple cancers, FGFR inhibitors (FGFRi) were supposed to treat patients with malignant tumors. Erdafitinib, a selective targeted FGFR inhibitor, has been approved in the treatment of advanced bladder cancer with FGFR gene mutation, as well as for clinical trials of metastatic breast cancer, prostate carcinoma, and other advanced solid tumors 17, 18. Here, we found that FGFRi also inhibited TNBC growth via reprogramming TIME, apart from its direct effect on tumor cells. FGFRs are dominantly expressed on CAFs in TME, and low-dose FGFRi suppressed the pro-tumor functions of CAFs without inhibiting tumor cell proliferation, indicating that regulating CAFs is another important potential mechanism for the anti-tumor activities of FGFRi.

VCAM-1 is well known for its up-regulated expression in activated endothelium, which helps adjust inflammation-associated vascular adhesion and the transendothelial migration of leukocytes. VCAM-1 was reported to be expressed on the surface of various cells such as macrophages, dendritic cells, fibroblasts, and tumor cells 22, In this study, we showed that VCAM-1 is relatively high expressed on CAFs in TME. Increasing evidence indicates that VCAM-1 is closely linked to the progression of several immunological disorders including rheumatoid arthritis, transplant rejection, asthma, and cancer 22. It was demonstrated that VCAM-1 represented a new mechanism of immune evasion, promoting T cell exclusion in tumors 23. Consistently, we here found that blocking FGFR signaling pathway markedly down-regulated VCAM-1 expression in CAFs, which was involved in the anti-tumor immunity by FGFR blockade. Together, these studies suggest that VCAM-1 might be a potential target for cancer immunotherapy.

Recently, accumulating studies focus on the non-redundant combination therapies to overcome immunotherapy resistance and broaden the clinical utility of ICT 7, 29, 30. To be noted, clarifying the context-dependent mechanism of treatment resistance and developing targeted therapies would be the key to the success of combination therapy. In this study, we revealed that the activation of FGFR signaling pathways might be a critical factor limiting ICT efficacy in immune-excluded TNBC. FGFR blockade not only significantly promoted T cell infiltration, but also enhanced the infiltration of other anti-tumor immune cells including NK cell and M1 macrophages, and further boosted the anti-tumor activity of CTL, which all help improve the response to ICT. As the absence of T cells in the tumor parenchyma occurs in most TNBC, these findings indicate that FGFR inhibitors might be a very promising therapeutic approach in combination with ICT in clinical practice.

## Experimental Section

*Study design*: This study was designed to uncover the key factors limiting T cell infiltration in TNBC and explore the therapeutic potential of targeting the key factors to enhance immunotherapy. Specifically, our primary objective was to investigate the relation between FGFR (derived from an unbiased bioinformatics screen based on TCGA) and T cell exclusion in TNBC, and we explored the mechanism of action and efficacy of FGFR blockade on anti-tumor immunity. All human sample collection and study protocols were approved by the Research Ethics Committee of the First Affiliated Hospital of Chongqing Medical University. For* in vivo* experiments, sample size for animal studies was based on statistical analysis of variance and previous experience with similar *in vivo* studies, and it was listed in the corresponding figure legends or on the figures. Animals were randomly assigned to treatment groups. Most experiments were independently replicated two or more times. The investigators were not blinded during data collection and analysis. Pathological analyses were performed in a blinded fashion.

*Bioinformatics:* The classification of tumor immune microenvironment in TNBC from TCGA was conducted as described previously 14. Briefly, the brisk diffuse type was classified as immune-inflamed type, and the brisk band-like type was classified as immune-excluded type. We compared the differential signaling pathways between immune-excluded and immune-inflamed TNBC by Gene Set Variation Analysis (GSVA). GSVA was performed by the GSVA package (version 3.10) of R (version 3.6.2). Gene sets of Reactome pathway database (version 6.2) were used for enrichment analysis. To explore the biological mechanisms of the FGFRs, Kyoto Encyclopedia of Genes and Genomes (KEGG) enrichment analysis was conducted by the “clusterProfiler” package in R. Critical pathways enriched in FGFR1-4 were identified. The CIBERSORT and MCPcounter algorithms were performed to evaluate the 23 types of stromal cells of TME in each group 31, 32. The GSEA analysis was performed by GSEA software 4.0.3. The Seurat R package was used for scRNA data analysis 33. GSE114727 scRNA data was selected from GSE114727_in Drop. Only terms with *p*<0.05 and the number of enriched genes ≥3 were considered statistically significant.

*Cell culture:* Murine mammary carcinoma cell lines 4T1 and EMT6, a mouse embryo fibroblast cell line NIH/3T3, and human TNBC cell lines MDA-MB-231 and BT-549 were obtained from American Type Culture Collection (ATCC) and cultured according to ATCC guidelines.

*Mice:* BALB/c and athymic nude mice were purchased from Laboratory Animal Center of Chongqing Medical University. All mouse protocols and experiments were performed in accordance with the Care and Use of Laboratory Animals and were approved by the Research Ethics Committee of the First Affiliated Hospital of Chongqing Medical University.

*Human Samples:* We obtained the tumor samples from TNBC patients (from 2011 to 2017) who had undergone a mastectomy before therapy from the First Affiliated Hospital of Chongqing Medical University. The use of pathological specimens, as well as the review of all pertinent patient records, was approved by the Research Ethics Committee of the First Affiliated Hospital of Chongqing Medical University (Project approval NO. 2017-012).

*Tumor inductions and treatment experiments:* For 4T1 and EMT6 models, 2×10^5^ tumor cells were orthotopically injected into BALB/c or athymic nude mice. Treatments were given as single agents or in combination, with the following regimen for each drug. FGFR inhibitor Erdafitinib (Selleck, #JNJ-42756493) treatment was initiated on day 7 after tumor inoculation and administered by oral gavage once every other day at 12.5 mg/kg. Anti-PD-1 antibody (clone RMP1-14, Bio X Cell, 10 mg/kg) was injected intraperitoneally on days 7, 10, 13, and 16 after tumor inoculation. For *in vivo* CD8^+^ T-cell depletion, mice were treated with 200 μg of anti-CD8 antibody every 4 days starting at 3 days before 4T1 tumor inoculation. For *in vivo* co-transplantation of fibroblasts and tumor cells, 4T1 tumor cells mixed with 3T3 fibroblasts at a ratio of 1:5 were orthotopically injected into BALB/c mice. Tumor size was measured by calipers every two or three days when tumors were palpable, and the volume was calculated using the formula V=as (width^2^×length)/2.

*Isolation of tumor-infiltrating cells:* Tumor samples were split with scissors and then subjected to enzymatic digestion with 2 mg/mL collagenase A (Roche) in DMEM for 45 min at 37 °C. Tissues were then filtered through 70-µm filters (BD Biosciences) to attain single-cell suspensions. After red blood cell lysis, all samples were washed and re-suspended in flow cytometry buffer for further use.

*Isolation of primary CAFs*: This experiment was done as previously described 34. Tumor samples were split with scissors and then subjected to enzymatic digestion with 2 mg/mL collagenase A (Roche) in DMEM for 45 mins at 37 °C. Tissues were then filtered through 70-µm filters (BD Biosciences) to attain single-cell suspension. The cells were cultured with DMEM containing 10% fetal bovine serum FBS and 1% penicillin and streptomycin. Cells that were adherent within 15 mins were considered as CAFs, while non-adherent cells were discarded. To acquire purer CAFs populations, we used magnetic-activated cell sorting (MASC) with anti-FSP (fibroblast specific protein) to purify the primary CAFs isolated as indicated above.

*Flow cytometry staining and analysis:* Live cells were sub-gated by staining with Fixable Viability Dye eFluor 450 (eBioscience) for 15 mins at 4 °C. Cells were then pre-incubated with purified anti-CD16/32 antibody (clone 93, BioLegend) for 10 mins on ice to block Fc receptors. After one wash, cells were incubated with various combinations of the following antibodies. Primary antibodies to cell surface markers directed against CD45 (30-F11), CD3 (145-2C11), CD4 (RM4-5), CD8a (53-6.7), CD11b (M1/70), Gr-1 (RB6-8C5), F4/80 (BM8) were from BioLegend; For intracellular staining, cells were fixed, permeabilized using Foxp3/Transcription Factor Staining Buffer Set (eBioscience), and then stained with fluorochrome-conjugated antibodies to FOXP3 (MF-14) from BioLegend. For cytokine staining, cells were first stimulated with Cell Stimulation Cocktail (eBioscience) at 37 °C for 4-6 h, and then stained with anti-IFN-γ (XMG1.2) from BioLegend. The stained cells were acquired by a BD FACSCanto II Flow Cytometer using BD FACSDiva software (BD Biosciences), and data generated were processed using FlowJo software.

*Mass cytometry (CyTOF) and data analysis:* Live single cells from tumor tissues were collected as described above. Then, for CyTOF analysis, cells were incubated with 25 µM cisplatin for 1 min (viability staining) and subsequently stained with a metal-labeled monoclonal antibody cocktail against cell surface molecules. After treatment with the Fixation/Permeabilization Buffer (eBioscience), cells were further incubated with monoclonal antibody cocktails against intracellular proteins. The samples were analyzed using the CyTOF 2 instrument (Fluidigm) at Institute of Liver Diseases (Beijing You-an Hospital Affiliated with Capital University of Medical Sciences). All CyTOF files were normalized and manually gated in Cytobank software. Data were transformed using the cytofAsinh function before they were applied to the downstream analysis. Phenograph clustering analysis in the R cytofkit package was executed on pooled samples to automatically categorize underlying immune subsets. Heat-maps were produced based on the mean value for each marker in clusters. Cell frequency in each cluster was calculated as the assigned cell events divided by the total CD45^+^ cell events in the same sample. Antibodies used in the mass cytometry analysis were purchased from Fluidigm: 147Sm-anti-CD45, 142Nd-anti-CD4, 141Pr-anti-PD1, 143Nd-anti-CD11b, 144Nd-anti-Siglec F, 145Nd-anti-CD69, 146Nd-anti-CD206, 148Sm-anti-Tbet, 149Sm-anti-CD103, 151Eu-anti-CD68, 152Sm-anti-CD3e, 156Gd-anti-CD14, 159Tb-anti-F4/80, 160Dy-anti-CD62L, 161Dy-anti-Ki67, 162Dy-anti-Ly-6C, 165Ho-anti-Foxp3, 166Er-anti-CD19, 167Er-anti-GATA3, 169Tm-anti-CD152, 170Er-anti-NK1.1, 171Yb-anti-CD8a, 172Yb-anti-CD86, 173Yb-anti-CD117, 174Yb-anti-ly-6G/Ly-6C(Gr-1), 175Lu-anti-I-A/I-E, 209Bi-anti-CD11c.

*Immunohistochemistry (IHC):* The tumor tissues were fixed in 4% formaldehyde solution (pH 7.0) and subsequently embedded in paraffin. Immunohistochemical studies were performed using the standard streptavidin-peroxidase (SP) method with the UltraSensitive TM SP Kit (Maixin-Bio, Fujian, China) according to the manufacturer's instructions. Tumor specimens were stained using antibodies against α-SMA (Abcam, ab7817), CD3 (Abcam, ab5690) or FGFR1 (Cell Signaling Technology, #9740). Negative control was performed by replacing the primary antibody with PBS. Immunostained slides were blindly evaluated by a trained pathologist.

*Immunofluorescence (IF):* IF was performed on Formalin fixed paraffin embedded (FFPE) sections from TNBC samples. The slides were deparaffinized and hydrated following IHC protocols. The primary antibodies used for IF staining includes anti-CD3 antibody (Abcam, ab5690), anti-SMA antibody (Abcam, ab7817) anti-FGFR1 (Cell Signaling Technology, #9740) and anti-VCAM-1 antibody (Abcam, ab134047). DyLight 488- or DyLight 594-conjugated secondary antibodies against rabbit or mouse IgG were obtained from Thermo Fisher Scientific. Sections were first stained with primary antibodies overnight at 4 °C and incubated with secondary antibodies for 1 h at room temperature. Cell nuclei were stained using 4', 6-diamidino-2-phenylindole (DAPI; Invitrogen, D1306) at room temperature for 5 mins. Images were acquired with an LSM 800 Confocal Microscope system (Zeiss, Germany).

*T cell migration assay:* Spleens from BALB/c mice were harvested and filtered through a 40-µm cell strainer to generate single-cell suspension. After red blood cell lysis, splenocytes were counted and seeded in complete RPMI 1640 medium supplemented with 50 µM β-mercaptoethanol and 10 mM HEPES onto 12-well plates coated with 2.5 µg/mL anti-CD3 (clone 145-2C11, BioLegend) and 3 µg/mL anti-CD28 (clone 37N, BioLegend) antibodies for the next 72 h activation. The T cell migration assay was conducted with transwells (8 μm pore size, 24-well plate, BD Biosciences, Billerica, MA, USA). Activated splenic T cells cultured in RPMI medium without fetal calf serum (FBS) were placed into the top chamber of the transwell, and migration-inducing medium (with 10% FBS) was added in the bottom chamber. To investigate the effect of CAFs on T cell migration, mouse CAFs would be cultured in the top chamber 1 h before adding T cell to this chamber. Recombinant VCAM-1 protein (ab276780) was used at a concentration of 10 µM. Cells were allowed to migrate for 6 h and then cells from the bottom chamber were harvested, labeled with CD4 (RM4-5) and CD8 (53-6.7) antibody and counted for 1 min using flow cytometry.

*Cytokine antibody array:* Mouse cytokine antibody array (Raybiotech, AAM-CYT-3) was used according to the manufacturer's instructions. Briefly, each captured antibody was printed on the membrane, and then treated or untreated cell lysate was added to the antibody array membranes. After extensive washing, the membranes were incubated with a cocktail of biotin-conjugated antiapoptotic protein antibodies. After incubation with HRP-streptavidin, the signals were visualized by chemiluminescence. The relative expression levels of target proteins were determined by comparing the signal intensities quantified by densitometry. Positive control was used to normalize the results from the different membranes.

*Enzyme-linked immunosorbent assay (ELISA):* The levels of VCAM-1 in cell culture supernatant and tumor tissues were detected by VCAM-1 ELISA kits (Abcam, ab201278 and ab223591) according to the manufacturer's instruction.

*Western blot:* Western blot was done as previously described 35. The following primary antibodies were used: VCAM-1 (Abcam, ab134047), p44/42 MAPK (Erk1/2) (Cell Signaling Technology, #4695), and p-p44/42 MAPK (Erk1/2) (Cell Signaling Technology, #4370).

*Generation of stable cells using lentiviral infection:* Mouse VCAM-1-targeting shRNAs were purchased from Genecopoeia Company. For lentiviral production, the lentiviral expression vector was co-transfected with lentivirus packing vectors into 293T cells using LipoD293 DNA *in vitro* Transfection Reagent (SignaGen Laboratories). Then, 48-72 h after transfection, cancer cell lines were stably infected with viral particles.

*RNA sequencing and data analysis:* 4T1 tumor samples were collected and then total RNA was purified using Trizol (Invitrogen). RNA samples were sent to Novogene Company for library construction and sequencing. Genes with adjusted *p* values <0.05 found by the DESeq2 R package were designated as DEGs. Functional enrichment of mouse differential genes was obtained from the gene sets of Mouse Genome Informatics (MGI). Pathways with *p*-value < 0.05 were considered significant.

*Statistical analysis:* Prism 8.0 software (GraphPad) was used for statistical analysis. Analysis for significance was performed by one-way or two-way ANOVA when more than two groups were compared and by t-test when only two groups were compared. *p*<0.05 was considered statistically significant (*p<0.05, **p<0.01, ***p<0.001, ****p<0.0001). Mouse survival was evaluated using the Kaplan-Meier method and analyzed by the Mantel-Cox log-rank test. All experiments were performed at least twice, and n refers to biological replicates.

## Supplementary Material

Supplementary figures.

## Abbreviations

TNBC: triple-negative breast cancer
FGFR: fibroblast growth factor receptor
CAFs: cancer-associated fibroblasts
VCAM-1: vascular cell adhesion molecule 1
ICT: immune checkpoint blockade therapy
CTLA-4: cytotoxic T lymphocyte associated antigen-4
PD-1: programmed death-1
PD-L1: programmed death ligand-1
TMB: tumor mutation burden
CTL: cytotoxic T cell
HR: hormone receptor
ER: estrogen receptor
PR: progesterone receptor
GSVA: Gene Set Variation Analysis
KEGG: Kyoto Encyclopedia of Genes and Genomes
ATCC: American Type Culture Collection
FFPE: Formalin fixed paraffin embedded
ELISA: Enzyme-linked immunosorbent assay
MGI: Mouse Genome Informatics
TIME: tumor immune microenvironment
IHC: immunohistochemistry
TIDE: Tumor Immune Dysfunction and Exclusion
FGFRi: FGFR inhibitor
MDSC: myeloid-derived suppressor cells
TISCH: Tumor Immune Single-cell Hub
CCK-8: Cell Counting Kit-8
GSEA: gene set enrichment analysis
ECM: extracellular matrix
RTK: receptor tyrosine kinase
