# Supplementary material for: FGFR blockade boosts T cell infiltration into triple-negative breast cancer by regulating cancer-associated fibroblasts
Source: Theranostics. 2022 May 27;12(10):4564–80. doi: 10.7150/thno.68972 (PMC9254240; doi:10.7150/thno.68972)
Supplement: Supplementary file 1 — Supplementary figures. [file thnov12p4564s1.pdf]

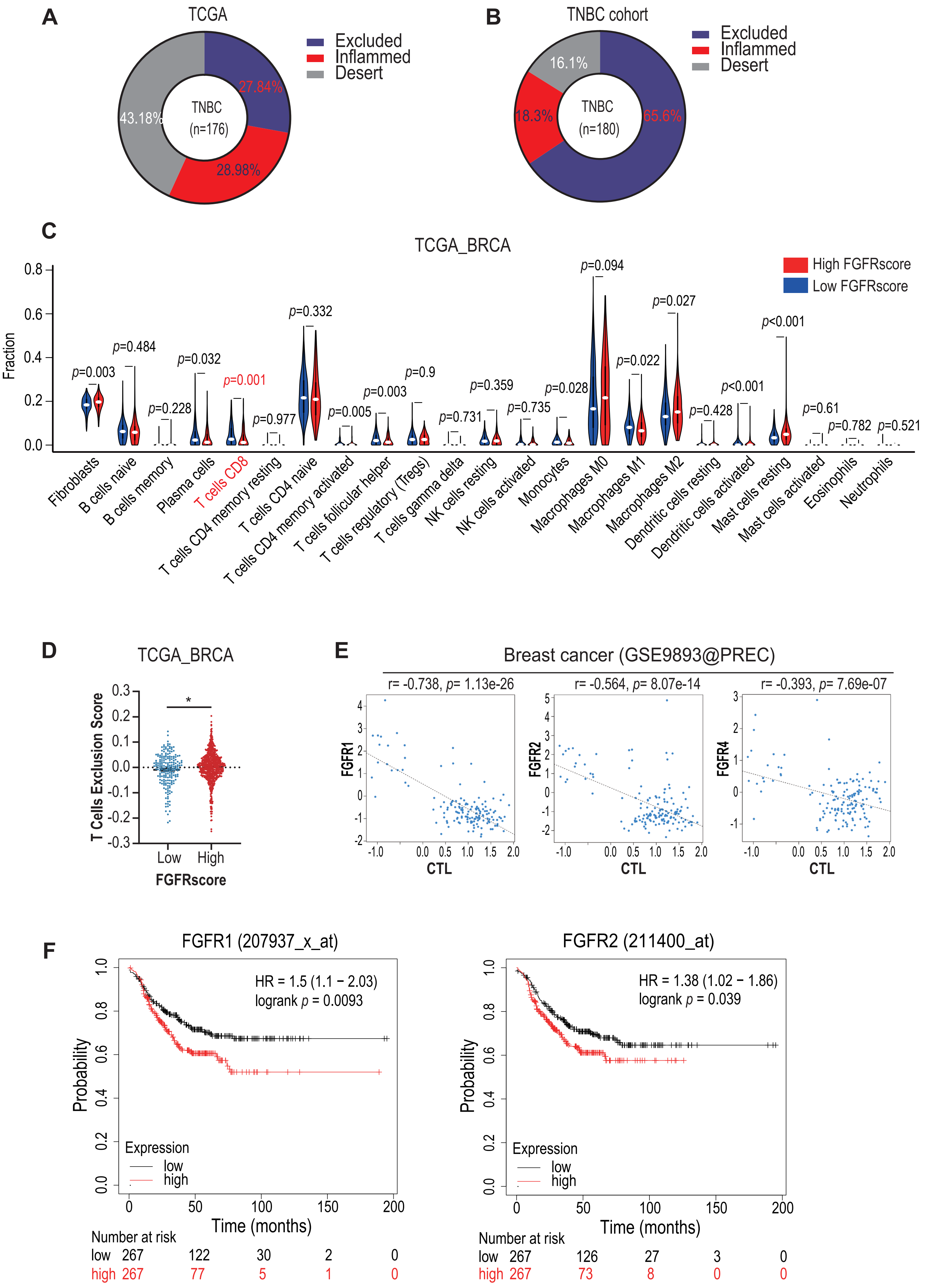
A
TCGA
TNBC
(n=176)
27.84%
28.98%
43.18%
Excluded
Inflammed
Desert
B
TNBC cohort
TNBC
(n=180)
16.1%
18.3%
65.6%
Excluded
Inflammed
Desert
C
TCGA_BRCA
High FGFRscore
Low FGFRscore
Fraction
p=0.003
p=0.484
p=0.228
p=0.032
p=0.001
p=0.977
p=0.332
p=0.005
p=0.003
p=0.9
p=0.731
p=0.359
p=0.735
p=0.028
p=0.094
p=0.022
p=0.027
p=0.428
p<0.001
p<0.001
p=0.61
p=0.782
p=0.521
Fibroblasts
B cells naive
B cells memory
Plasma cells
T cells CD8
T cells CD4 memory resting
T cells CD4 naive
T cells CD4 memory activated
T cells follicular helper
T cells regulatory (Tregs)
T cells gamma delta
NK cells resting
NK cells activated
Monocytes
Macrophages M0
Macrophages M1
Macrophages M2
Dendritic cells resting
Dendritic cells activated
Mast cells resting
Mast cells activated
Eosinophils
Neutrophils
D
TCGA_BRCA
T Cells Exclusion Score
*
Low
High
FGFRscore
E
Breast cancer (GSE9893@PREC)
r= -0.738, p= 1.13e-26
r= -0.564, p= 8.07e-14
r= -0.393, p= 7.69e-07
FGFR1
FGFR2
FGFR4
CTL
F
FGFR1 (207937_x_at)
HR = 1.5 (1.1 − 2.03)
logrank p = 0.0093
Probability
Expression
low
high
Time (months)
Number at risk
low 267 122 30 2 0
high 267 77 5 1 0
FGFR2 (211400_at)
HR = 1.38 (1.02 − 1.86)
logrank p = 0.039
Probability
Expression
low
high
Time (months)
Number at risk
low 267 126 27 3 0
high 267 73 8 0 0

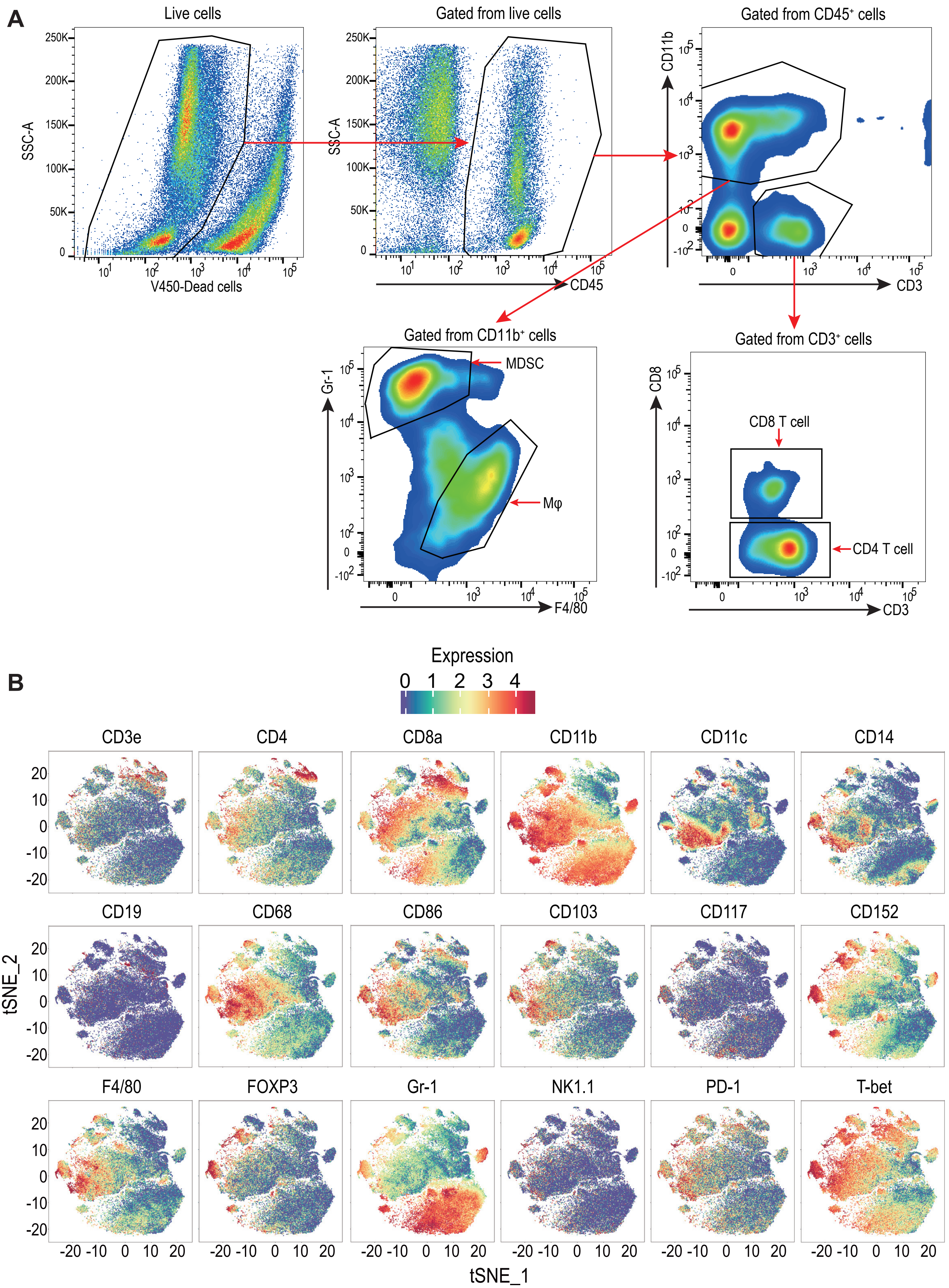

A
Live cells
SSC-A
V450-Dead cells
Gated from live cells
CD45
Gated from CD45+ cells
CD11b
CD3
Gated from CD11b+ cells
Gr-1
F4/80
MDSC
Mφ
Gated from CD3+ cells
CD8
CD8 T cell
CD4 T cell
B
Expression
CD3e
CD4
CD8a
CD11b
CD11c
CD14
CD19
CD68
CD86
CD103
CD117
CD152
F4/80
FOXP3
Gr-1
NK1.1
PD-1
T-bet
tSNE_2
tSNE_1

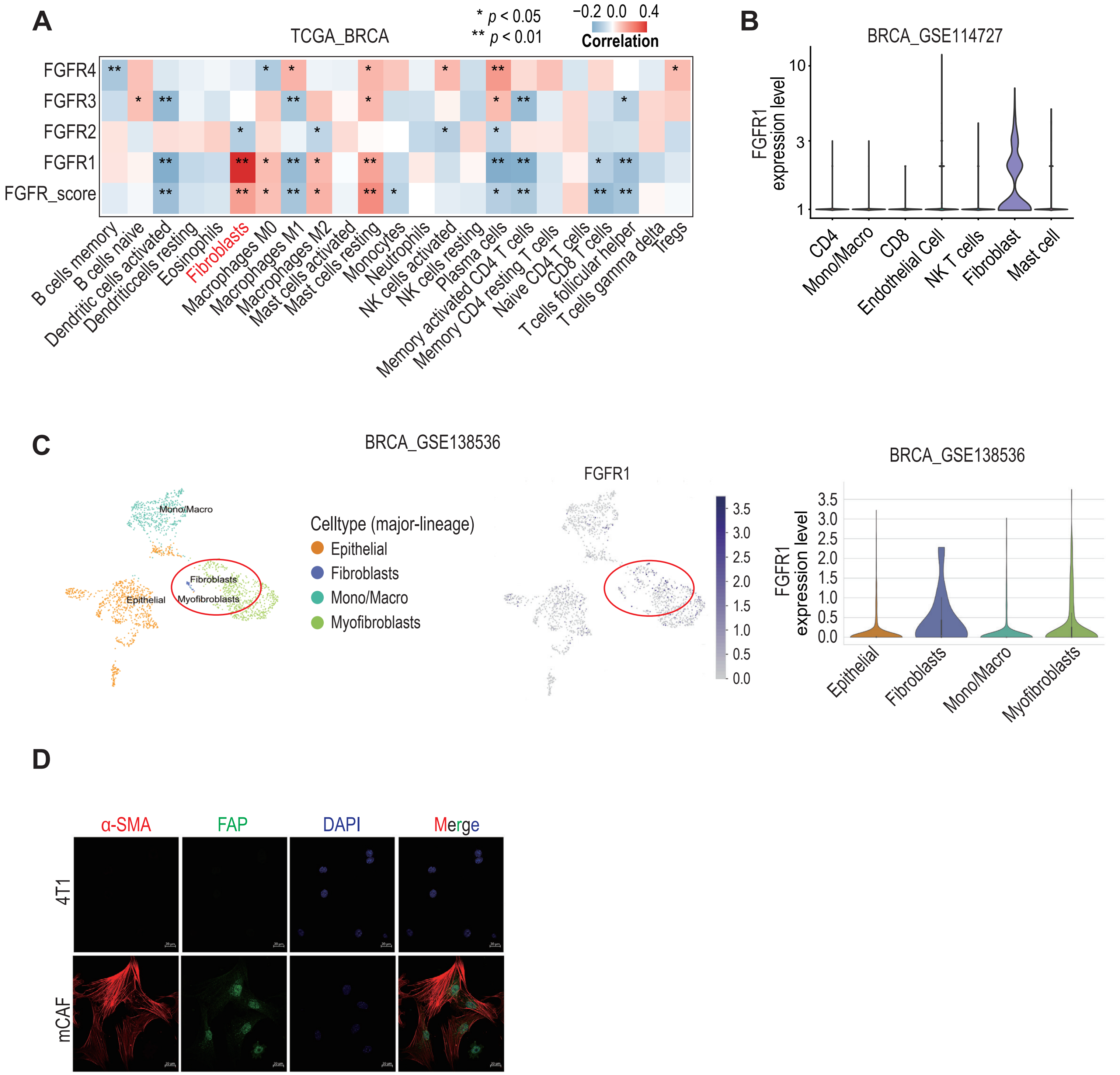
A
TCGA_BRCA
* p < 0.05
** p < 0.01
−0.2 0.0 0.4
Correlation
FGFR4
FGFR3
FGFR2
FGFR1
FGFR_score
B cells memory
B cells naive
Dendritic cells activated
Dendriticcells resting
Eosinophils
Fibroblasts
Macrophages M0
Macrophages M1
Macrophages M2
Mast cells activated
Mast cells resting
Monocytes
Neutrophils
NK cells activated
NK cells resting
Plasma cells
Memory activated CD4 T cells
Memory CD4 resting T cells
Naive CD4 T cells
CD8 T cells
T cells follicular helper
T cells gamma delta
Tregs
B
BRCA_GSE114727
FGFR1 expression level
10
3
1
CD4
Mono/Macro
CD8
Endothelial Cell
NK T cells
Fibroblast
Mast cell
C
BRCA_GSE138536
Mono/Macro
Fibroblasts
Epithelial
Myofibroblasts
Celltype (major-lineage)
Epithelial
Fibroblasts
Mono/Macro
Myofibroblasts
FGFR1
3.5
3.0
2.5
2.0
1.5
1.0
0.5
0.0
BRCA_GSE138536
FGFR1 expression level
Epithelial
Fibroblasts
Mono/Macro
Myofibroblasts
D
α-SMA
FAP
DAPI
Merge
4T1
mCAF

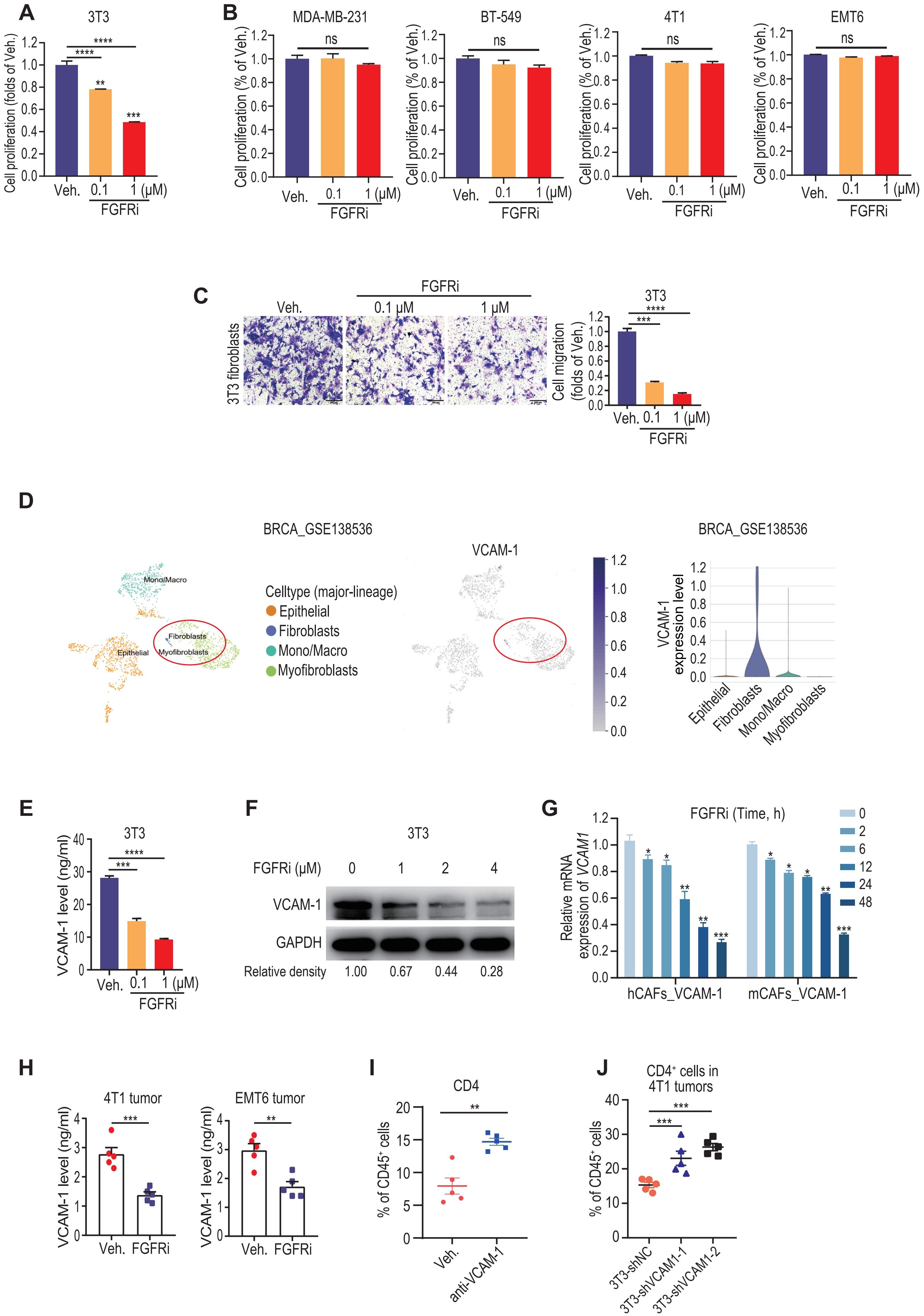

A
3T3
Cell proliferation (folds of Veh.)
Veh.
0.1
1 (μM)
FGFRi
B
MDA-MB-231
BT-549
4T1
EMT6
ns
Cell proliferation (% of Veh.)
C
FGFRi
Veh.
0.1 μM
1 μM
3T3 fibroblasts
Cell migration (folds of Veh.)
D
BRCA_GSE138536
Mono/Macro
Fibroblasts
Myofibroblasts
Epithelial
Celltype (major-lineage)
Epithelial
Fibroblasts
Mono/Macro
Myofibroblasts
VCAM-1
VCAM-1 expression level
E
VCAM-1 level (ng/ml)
F
FGFRi (μM)
0
1
2
4
VCAM-1
GAPDH
Relative density
1.00
0.67
0.44
0.28
G
FGFRi (Time, h)
Relative mRNA expression of VCAM1
hCAFs_VCAM-1
mCAFs_VCAM-1
0
2
6
12
24
48
H
4T1 tumor
EMT6 tumor
Veh.
FGFRi
I
CD4
% of CD45+ cells
anti-VCAM-1
J
CD4+ cells in 4T1 tumors
3T3-shNC
3T3-shVCAM1-1
3T3-shVCAM1-2

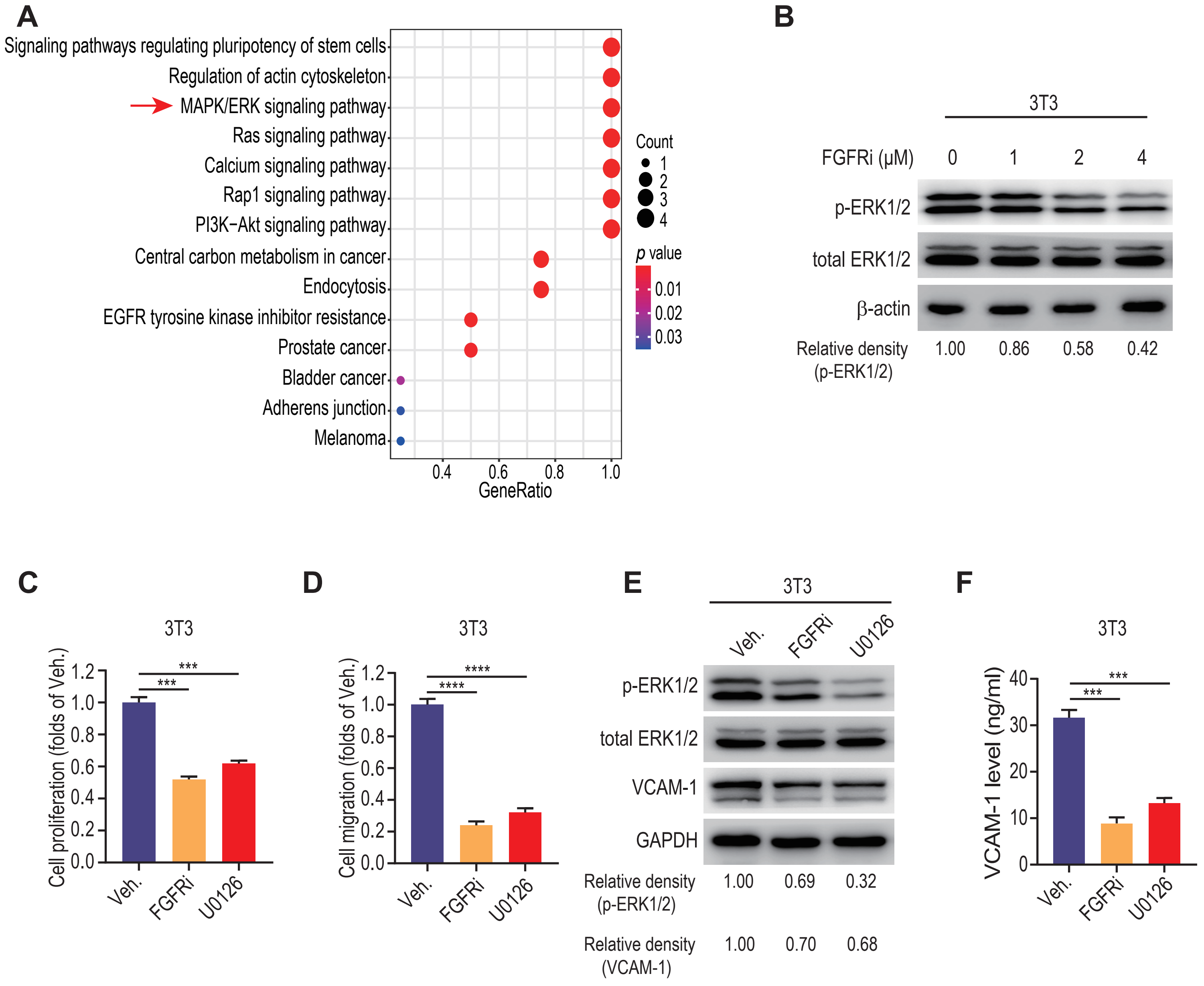

A
Signaling pathways regulating pluripotency of stem cells
Regulation of actin cytoskeleton
MAPK/ERK signaling pathway
Ras signaling pathway
Calcium signaling pathway
Rap1 signaling pathway
PI3K−Akt signaling pathway
Central carbon metabolism in cancer
Endocytosis
EGFR tyrosine kinase inhibitor resistance
Prostate cancer
Bladder cancer
Adherens junction
Melanoma
0.4 0.6 0.8 1.0
GeneRatio
Count
1 2 3 4
p value
0.01 0.02 0.03
B
3T3
FGFRi (μM) 0 1 2 4
p-ERK1/2
total ERK1/2
β-actin
Relative density (p-ERK1/2) 1.00 0.86 0.58 0.42
C
3T3
Cell proliferation (folds of Veh.)
***
***
Veh. FGFRi U0126
D
3T3
Cell migration (folds of Veh.)
****
****
Veh. FGFRi U0126
E
3T3
Veh. FGFRi U0126
p-ERK1/2
total ERK1/2
VCAM-1
GAPDH
Relative density (p-ERK1/2) 1.00 0.69 0.32
Relative density (VCAM-1) 1.00 0.70 0.68
F
3T3
VCAM-1 level (ng/ml)
***
***
Veh. FGFRi U0126

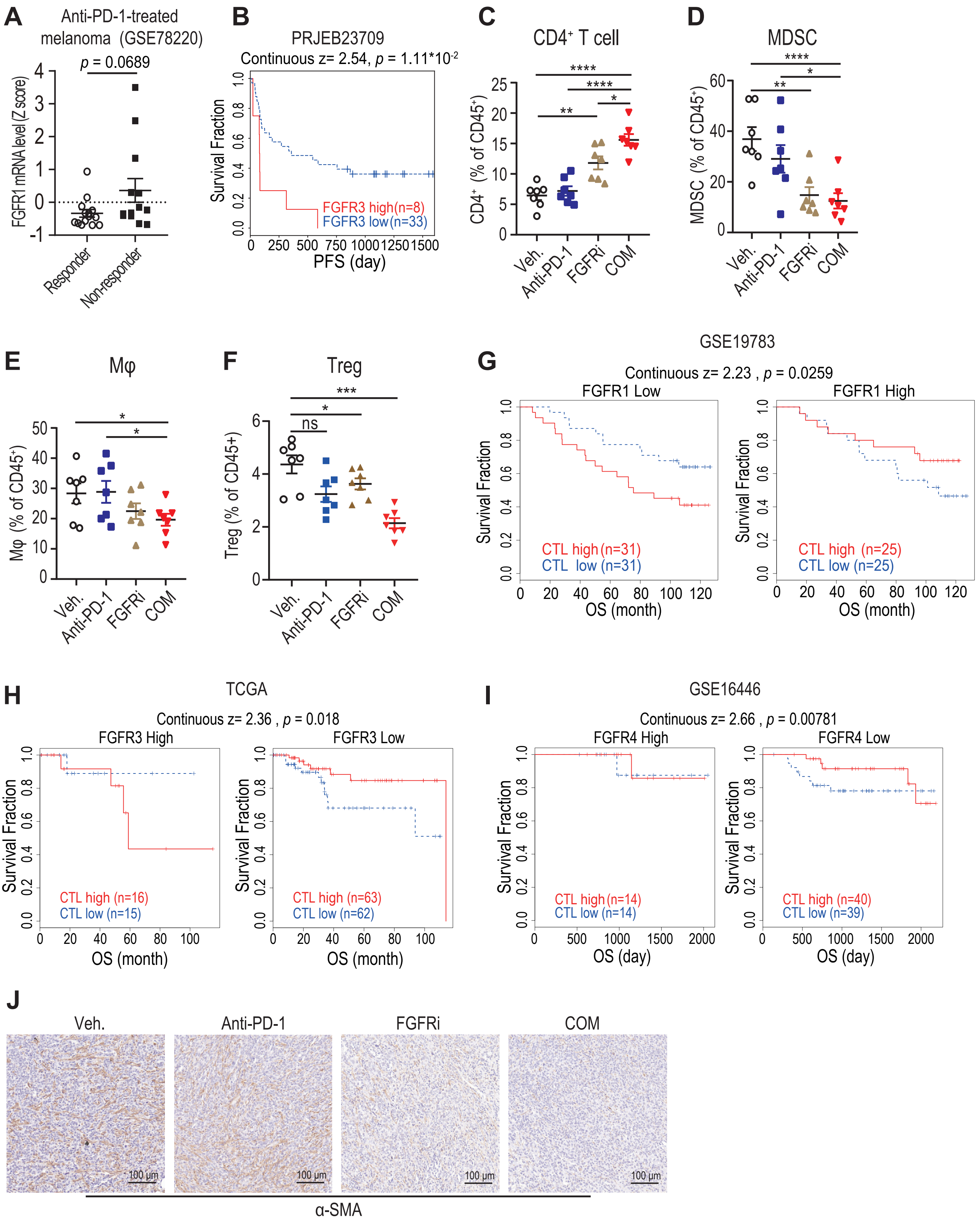

A
Anti-PD-1-treated melanoma (GSE78220)
p = 0.0689
FGFR1 mRNA level (Z score)
Responder
Non-responder
B
PRJEB23709
Continuous z= 2.54, p = 1.11*10-2
Survival Fraction
PFS (day)
FGFR3 high(n=8)
FGFR3 low(n=33)
C
CD4+ T cell
CD4+ (% of CD45+)
Veh.
Anti-PD-1
FGFRi
COM
D
MDSC
MDSC (% of CD45+)
E
Mφ
Mφ (% of CD45+)
F
Treg
Treg (% of CD45+)
ns
G
GSE19783
Continuous z= 2.23 , p = 0.0259
FGFR1 Low
FGFR1 High
OS (month)
CTL high (n=31)
CTL low (n=31)
CTL high (n=25)
CTL low (n=25)
H
TCGA
Continuous z= 2.36 , p = 0.018
FGFR3 High
FGFR3 Low
CTL high (n=16)
CTL low (n=15)
CTL high (n=63)
CTL low (n=62)
I
GSE16446
Continuous z= 2.66 , p = 0.00781
FGFR4 High
FGFR4 Low
OS (day)
CTL high (n=14)
CTL low (n=14)
CTL high (n=40)
CTL low (n=39)
J
Veh.
Anti-PD-1
FGFRi
COM
100 μm
α-SMA

Supplementary Figure legends

Figure S1. The correlation between FGFR1/2 expression and relapse-free survival (RFS) of patients with TNBC. A) Immune phenotypes of TNBC from TCGA database. B) Immune phenotypes of our TNBC cohort. C) Differences in 23 types of stromal cells from TME in BRCA of TCGA were compared after grouping by median FGFRscore. D) Differences in T cell exclusion score in BRCA of TCGA were compared after grouping by median FGFRscore. E) The correlation between FGFR1/2/4 expression and cytotoxic T lymphocytes (CTL) infiltration in breast cancer (GSE9893 dataset) based on Tumor Immune Dysfunction and Exclusion (TIDE) system. F) The correlation between FGFR1/2 expression and relapse-free survival (RFS) of patients with TNBC from Kaplan-Meier (KM) Plotter database.

Figure S2. tSNE plot of TILs overlaid with the expression of indicated markers. A) Gating strategy to identify immune cell subsets in 4T1 tumor. B) tSNE plot of TILs overlaid with the expression of indicated markers.

Figure S3. FGFR1 expression in tumor microenvironment of breast cancer. A) The correlation between FGFRs and 23 types of stromal cells from TME in BRCA of TCGA. B) FGFR1 expression in TME of breast cancer (GSE114727). C) FGFR1 expression in TME of breast cancer from GSE138536 dataset. D) Representative images of α-SMA and FAP immunofluorescent staining in 4T1 cells and mouse CAFs.

Figure S4. Blocking FGFR pathway inhibited cell proliferation, migration and VCAM-1 secretion of CAFs. A) The effect of FGFRi Erdafitinib on cell proliferation of 3T3 fibroblasts for 48 h was detected by CCK-8 assay (n=3 biological replicates, one-way ANOVA). B) The effect of FGFRi Erdafitinib on cell proliferation of breast cancer cell lines for 48 h was detected by CCK-8 assay. C) The effect of FGFRi Erdafitinib on cell migration of 3T3 fibroblasts was detected by transwell migration assay (n=3 biological replicates, one-way ANOVA). D) VCAM-1 expression in TME of breast cancer from GSE138536 dataset based on TISCH system. E) The effect of FGFRi Erdafitinib on VCAM-1 level in cell supernatant of 3T3 fibroblasts was detected by ELISA (n=3 biological replicates, one-way ANOVA). F) The effect of FGFRi Erdafitinib on VCAM-1 expression of 3T3 fibroblasts was detected by western blot. G) The effect of different durations of FGFRi Erdafitinib on VCAM-1 mRNA expression in human CAFs and mouse CAFs was examined by qPCR. H) The effect of FGFRi Erdafitinib on VCAM-1 level in tumor extract of 4T1 and EMT6 tumors was detected by ELISA (n=3 biological replicates, one-way ANOVA). I) $CD4^+$ T cell population in primary 4T1 tumors from mice treated with vehicle or anti-VCAM1 (n=5, one-way ANOVA). J) Percentages of $CD4^+$ T cells in primary 4T1 tumors from mice treated with indicated therapies (n=5, one-way ANOVA).

Figure S5. The activation of MAPK/ERK pathway maintains FGFRs function in CAFs *in vitro*. A) GSEA (Reactome pathway analysis) of FGFR1-4 in breast cancer from GEO. B) The effect of FGFRi Erdafitinib on p-ERK1/2 and total ERK1/2 expression of 3T3 fibroblasts was detected by western blot. C) The effect of MAPK pathway inhibitor U0126 on cell proliferation of and 3T3 fibroblasts for 48 h was detected by CCK-8 assay (n=3 biological replicates, one-way ANOVA). D) The effect of U0126 on cell migration of 3T3 fibroblasts was detected by transwell migration assay (n=3 biological replicates, one-way ANOVA). E) The effect of U0126 on VCAM-1 expression of 3T3 fibroblasts was detected by western blot. F) The effect of U0126 on VCAM-1 level in cell supernatant of 3T3 fibroblasts was detected by ELISA (n=3 biological replicates, one-way ANOVA).

Figure S6. High expression of FGFRs is correlated with poor survival of ICT-treated patients and T cell dysfunction. A) FGFR1 mRNA expression in pre-treatment tumors of responder (n=14) *versus* non-responder (n=13) melanoma patients (t-test). B) Progression-free survival of melanoma patients who had high FGFR3 *versus* low FGFR3 expressed in the tumors before anti-PD-1 treatment (PRJEB23709). C-F) Percentages of $CD4^+$ T cells, $CD8^+$ T cells, MDSC and Mφ in primary 4T1 tumors from mice treated with indicated therapies (n=6, one-way ANOVA). G-I) Kaplan-Meier survival analysis of low CTL (blue) *versus* high CTL (red) in FGFRs-high or FGFRs-low breast cancer. J) Representative α-SMA staining in indicated therapy-treated 4T1 tumors.
